# Conserved metabolic regulator ArcA responds to oxygen availability, iron limitation, and cell envelope perturbations during bacteremia

**DOI:** 10.1128/mbio.01448-23

**Published:** 2023-09-08

**Authors:** Aric N. Brown, Mark T. Anderson, Sara N. Smith, Michael A. Bachman, Harry L. T. Mobley

**Affiliations:** 1 Department of Microbiology and Immunology, University of Michigan Medical School, Ann Arbor, Michigan, USA; 2 Department of Pathology, University of Michigan Medical School, Ann Arbor, Michigan, USA; Georgia Institute of Technology, Atlanta, Georgia, USA

**Keywords:** bacteremia, two-component regulatory systems, metabolic regulation, Gram-negative pathogenesis

## Abstract

Gram-negative facultative anaerobes often cause bacteremia, a systemic infection associated with severe clinical outcomes. ArcAB, a two-component regulatory system that represses aerobic respiration, is a key mediator of metabolic adaptation for such bacteria. Using targeted mutational analysis informed by global genetic screens, we identified the *arcA* gene as promoting fitness of *Citrobacter freundii*, *Klebsiella pneumoniae*, and *Serratia marcescens* but not *Escherichia coli* in a murine model of bacteremia. *arcA* mutants exhibit a dysregulated response to changes in oxygen availability, iron limitation, and membrane perturbations, which bacterial cells experience during infection. The genetic response of the *arcA* mutants to the cationic antimicrobial peptide polymyxin B supports an expanded role for ArcA as an activator in response to membrane damage. ArcA function is linked to electron transport chain activity based on its response to proton motive force uncoupling by carbonylcyanide-*m*-chlorophenylhydrazone (CCCP). Differences in lactate, acetate, and lactate dehydrogenase activity between *arcA* mutant and wild-type cells following CCCP treatment support an ArcA-mediated shift to fermentation independent of oxygen availability. This study highlights the semi-conserved role of ArcA during bacteremia and consolidates infection phenotypes into a comprehensive model based on respiratory activity.

**IMPORTANCE:**

Infections of the bloodstream are life-threatening and can result in sepsis. Gram-negative bacteria cause a significant portion of bloodstream infections, which is also referred to as bacteremia. The long-term goal of our work is to understand how such bacteria establish and maintain infection during bacteremia. We have previously identified the transcription factor ArcA, which promotes fermentation in bacteria, as a likely contributor to the growth and survival of bacteria in this environment. Here, we study ArcA in the Gram-negative species *Citrobacter freundii*, *Klebsiella pneumoniae,* and *Serratia marcescens*. Our findings aid in determining how these bacteria sense their environment, utilize nutrients, and generate energy while countering the host immune system. This information is critical for developing better models of infection to inform future therapeutic development.

## INTRODUCTION

Metabolic flexibility is an established characteristic of opportunistic bacteria and may be a prerequisite for transitioning between non-pathogenic and pathogenic environments. Facultatively anaerobic bacteria are capable of respiration and fermentation and are the most commonly isolated pathogens from Gram-negative bacteremia patients (1, 2). However, the factors dictating metabolic shifts throughout infection, including during colonization and dissemination, are poorly understood. *Citrobacter freundii*, *Escherichia coli*, *Klebsiella pneumoniae*, and *Serratia marcescens* cause many community and hospital-acquired cases of bacteremia (3). Sepsis, the single highest cause of in-hospital mortality in the United States, commonly results from bacteremia (4). *E. coli* and *K. pneumoniae* are the two most frequently isolated Gram-negative pathogens in sepsis cases, while *C. freundii* and *S. marcescens* are emerging bacteremia pathogens of increasing concern (5 – 8). The long-term goal of this work is to advance our understanding of the metabolic and regulatory pathways that these bacteria employ within the host bloodstream.

Our group previously utilized *C. freundii*, *E. coli*, *K. pneumoniae*, and *S. marcescens* transposon mutant libraries and TnSeq to identify genes critical to bacteremia (9 – 12). Genes encoding pathways of central carbon metabolism were among the significant fitness genes shared between species. Understanding the regulation of such processes is critical for establishing comprehensive models of pathogenesis (13). The TnSeq results were compared to identify shared transcriptional regulators of central metabolism contributing to bacterial fitness. Interruption of genes encoding the two-component system ArcAB resulted in a significant loss of fitness for *C. freundii*, *K. pneumoniae*, and *S. marcescens* but not *E. coli*. The response regulator ArcA is a transcription factor (14) that regulates aerobic and anaerobic transitions in *E. coli* in coordination with FNR, IHFA-B, CRP, and Fis (15). ArcA was the only such regulator from this group that was implicated in bacteremia fitness across *C. freundii*, *K. pneumoniae*, and *S. marcescens*. ArcA is already known to be employed by *Haemophilus influenzae* and *Salmonella enterica* in systemic infections (16, 17). The most well-studied function of ArcA is repression of aerobic respiration pathways, including the citric acid cycle. Global regulation of metabolism by ArcA is critical for balancing catabolic efficiency (energy production) with fueling anabolism (biomass growth) (18 – 20). ArcA and its cognate sensor kinase ArcB function in conditions where oxygen utilization decreases (21) and in response to reactive oxygen species (22). Global regulators integrate multiple stimuli for metabolic reprogramming (19, 23), and several signals in the infection environment likely impact ArcA activity. Here, the role of ArcA in repressing respiration in the mammalian bloodstream is examined.

## RESULTS

### Conservation of ArcA

**Fig 1:**
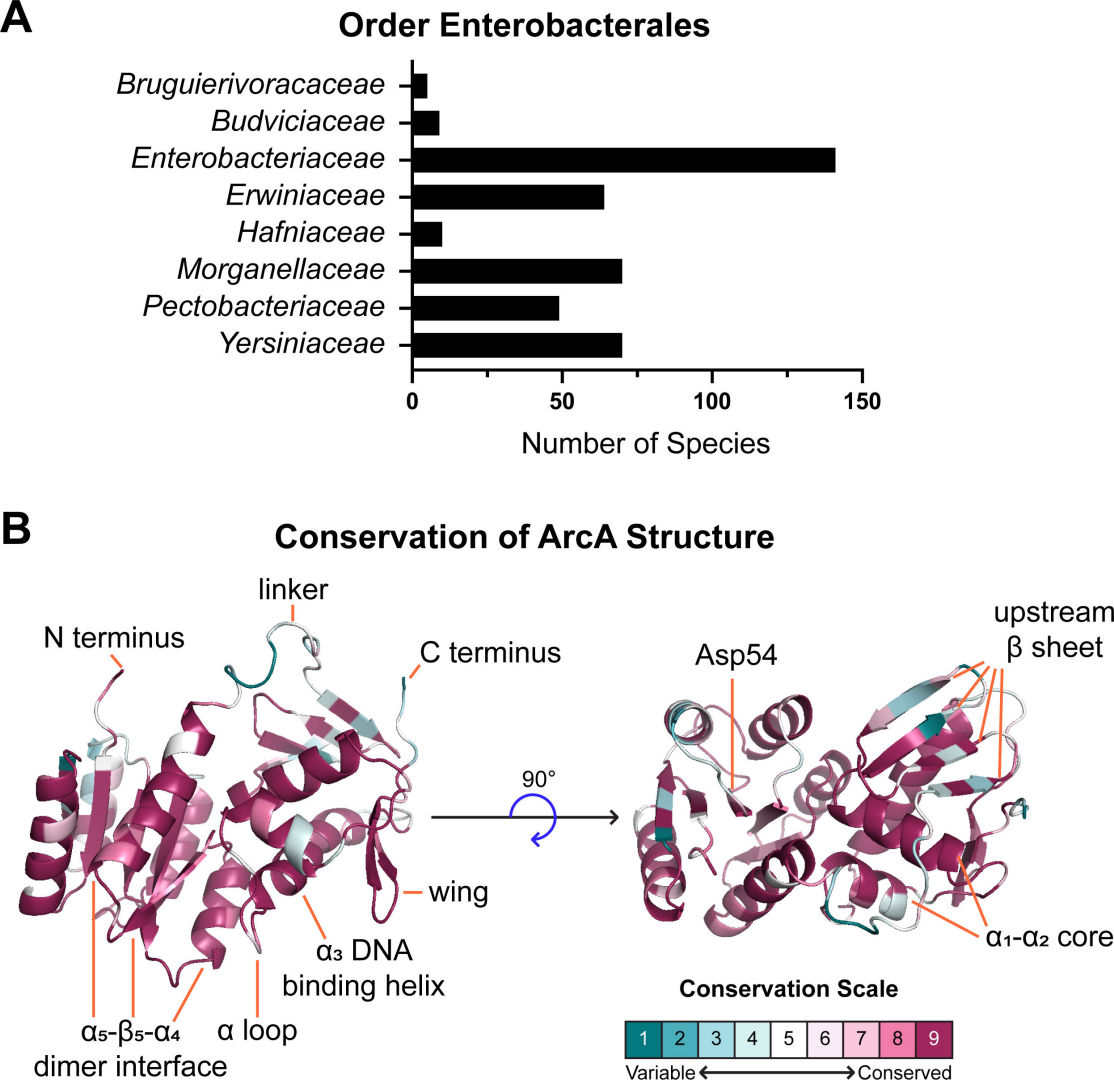
ArcA is structurally conserved across order *Enterobacterales*. (**A**) Four hundred nineteen ArcA amino acid sequences of 418 species across 8 families in order *Enterobacterales* were identified with BV-BRC (31) and aligned (File S1). (**B**) The multi-sequence sequence alignment (32, 33) was mapped onto a structure of ArcA (34, 35) with Consurf (36) and visualized with pyMOL (37). The average grade of conservation for 238 residues on a scale of 1 to 9 was 7.7. The regions with the greatest variation in conservation are the linker domain and the upstream β sheet of the DNA-binding domain. ArcB activates ArcA via phosphorylation of Asp^54^ which is highly conserved among the species examined in addition to the DNA-binding helix and structures supporting it. Conservation of individual residues is visualized in Fig. S1.

ArcA conservation was assessed across order *Enterobacterales* by mapping protein sequences with Consurf (24) to an Alpha Fold-predicted structure of ArcA (25, 26). Four-hundred nineteen ArcA amino acids sequences (File S1) from 418 species across 8 families were identified in total (Fig. 1A) with 150 unique sequences remaining after identical sequence removal. Conservation analysis based on ArcA structure and sequence phylogeny calculated an average pairwise distance of 0.07, meaning approximately 7% of residues differ between any two ArcA sequences. On a scale of 1 to 9, the average conservation level of the 238 residues was 7.7, and more than 75% of residues scored in the “conserved” range of 6 to 9 (Fig. S1). The N-terminal receiver domain of ArcA was very well conserved including the 54th residue aspartate phosphorylated by ArcB (27) (Fig. 1B). In contrast, the linker domain directly following the receiver domain was one of the least conserved regions. The C-terminal winged helix-turn-helix (wHTH) DNA-binding domain is broadly maintained in this model of ArcA (28, 29). Such OmpR-like wHTH regulators are characterized by an antiparallel β-sheet on the -terminal side of the N-terminal side of the binding domain that likely determines binding specificity (28), and lower conservation of the β-sheet here suggests potential species-based differences in DNA-binding capabilities. In concordance with the larger sequence comparison, ArcA homology in clinical strains of *C. freundii*, *E. coli*, *K. pneumoniae*, and *S. marcescens* ranged from 93.70% to 99.58% amino acid identity (Fig. S2) (30). Structural conservation coupled with the previous genetic screens prompted investigation of a shared role for ArcA during bloodstream infections.

### Contribution of *arcA* to fitness in murine bacteremia model

**Fig 2:**
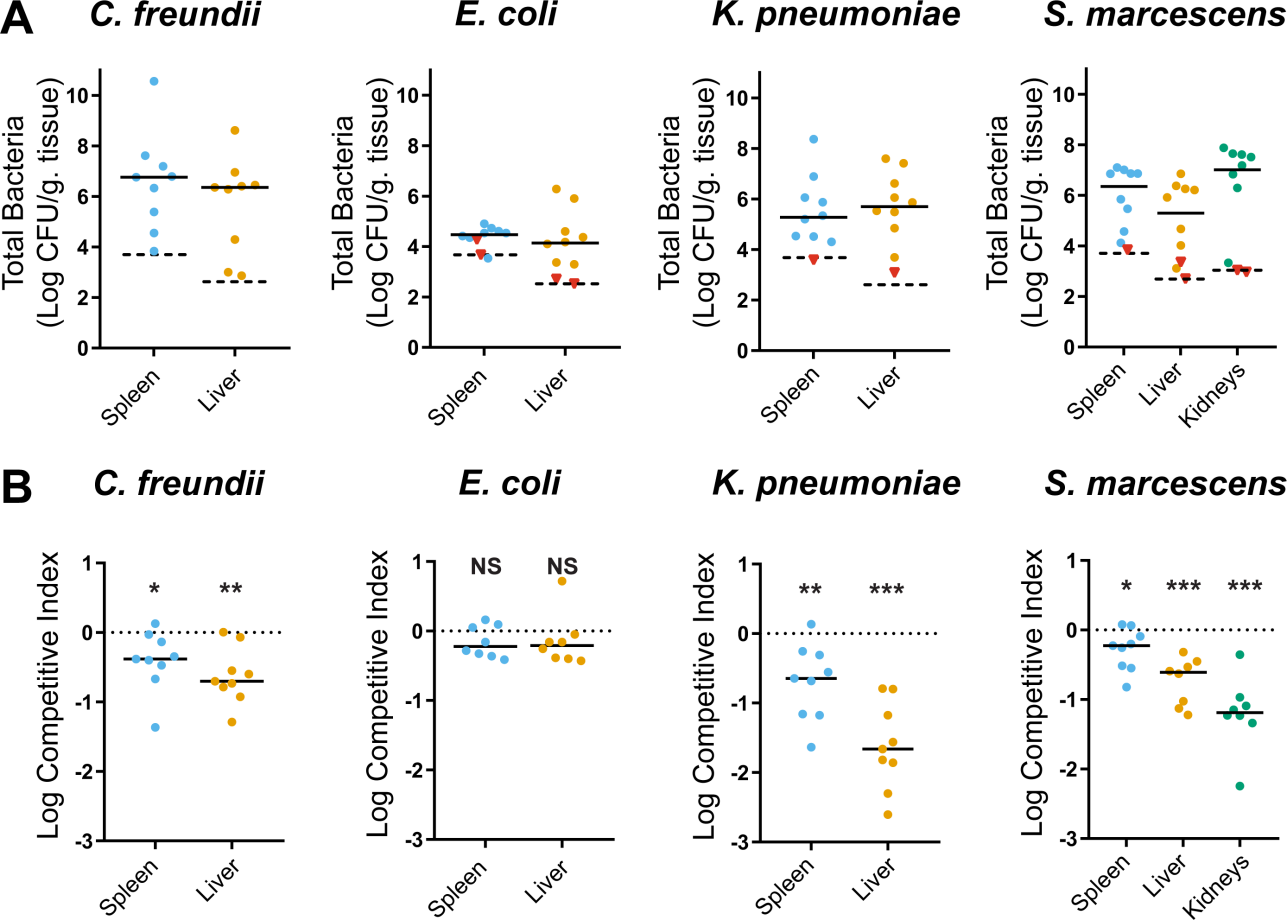
*arcA* encodes a fitness factor in a murine model of bacteremia. Wild-type (WT) strains and Δ*arcA* mutant constructs were cultured to mid-log phase in LB. Cells were washed in PBS and mixed 1:1 to prepare the inoculum for each species at an average target total CFU of 1 × 10^8^ (*C. freundii*), 1 × 10^5^ (*K. pneumoniae*), 1 × 10^7^ (*Serratia marcescens*), and 2 × 10^6^ (*E. coli*). Mice were sacrificed 24 h post tail vein inoculation, and organs were harvested and plated on LB with and without antibiotics for differential CFU enumeration. (**A**) Total CFU was normalized to tissue weight for all organs. The limit of detection is denoted as a dashed black line, and red triangles are samples not included in calculating competitive indices due to limited CFU recovery. (**B**) Competitive indices (CI) were calculated by dividing the ratio of *arcA* mutant counts to WT counts in the inoculum (input) to that in the organs (output). Dots in the burden and CI graphs represent the organ from one mouse, and median values are presented as solid horizontal lines. Significance of log transformed CI was determined via a one-sample *t*-test with a null hypothetical value of zero, represented as a dotted a line. *P*-values: *≤0.05, **≤0.01, ***≤0.001, NS = not significant.

Competition experiments between wild-type strains and *arcA* mutant constructs (Table 1) were conducted in a murine bacteremia model to assess how ArcA contributes to bacterial survival and replication, collectively referred to as fitness. Each species colonized the liver and spleen 24 h post inoculation (Fig. 2A). *S. marcescens* is the only species to reliably colonize kidneys based on our previous findings (38) and achieved high bacterial burdens again here. A significant *arcA*-dependent fitness defect was observed in the liver and spleen for *C. freundii*, *K. pneumoniae*, and *S. marcescens* (Fig. 2B). The largest fitness defect for *C. freundii* and *K. pneumoniae* was in the liver where *arcA* mutants were outcompeted 6.0-fold and 99.4-fold, respectively. The *S. marcescens arcA* mutant was most outcompeted in the kidneys (33.7-fold), indicating the link between ArcA and fitness in this model is organ-and species-specific. These results validate our previous TnSeq findings identifying fitness potential of ArcA among a substantial pool of transposon mutants (10 – 12). No significant fitness defect was observed for the *E. coli arcA* mutant in the spleen or the liver, a notable contrast to the other species. This finding is corroborated by earlier studies in which an *E. coli arcA* transposon mutant was not associated with a significant fitness defect in spleens (9, 39). Thus, although the ArcA sequence analysis demonstrates a high level of conservation, the fundamental contribution of *E. coli* ArcA to bacterial fitness during infection differs substantially from the other species. We, therefore, chose to explore *in vitro* how ArcA contributes to fitness during bacteremia in *C. freundii, K. pneumoniae,* and *S. marcescens*.

**TABLE 1:** Strains and constructs used in study

Species	Parent strain	Genotype	Description	Reference(s)
*C. freundii*	UMH14	Wild-type strain		Anderson et al. (11)
*C. freundii*	UMH14	Δ*arcA*	Δ*arcA*::*nptII* knock-out construct	This study
*C. freundii*	UMH14	Δ*arcA*::*arcA*	Reversion to wild-type allele	This study
*E. coli*	CFT073	Wild-type strain		Welch et al. (40), Mobley et al. (41)
*E. coli*	CFT073	Δ*arcA*	Δ*arcA*::*nptII* knock-out construct	This study
*K. pneumoniae*	KPPR1	Wild type		Broberg et al. (42)
*K. pneumoniae*	KPPR1	Δ*arcA*	Δ*arcA*::*nptII* knock-out construct	This study
*K. pneumoniae*	KPPR1	Δ*arcA +* pBBR1MCS-5	*arcA* knock-out construct with empty vector pBBR1MCS-5	This study
*K. pneumoniae*	KPPR1	Δ*arcA +* pBBR1MCS-5+*arcA*	Δ*arcA*::*nptII* knock-out construct complemented with pBBR1MCS-5 +*arcA* gene	This study
*S. marcescens*	UMH9	Wild-type construct		Anderson et al. (10)
*S. marcescens*	UMH9	Δ*arcA*	Δ*arcA*::*nptII* knock-out construct	This study
*S. marcescens*	UMH9	Δ*arcA::arcA*	Reversion to wild-type allele	This study

### *In vitro* growth analysis

**Fig 3:**
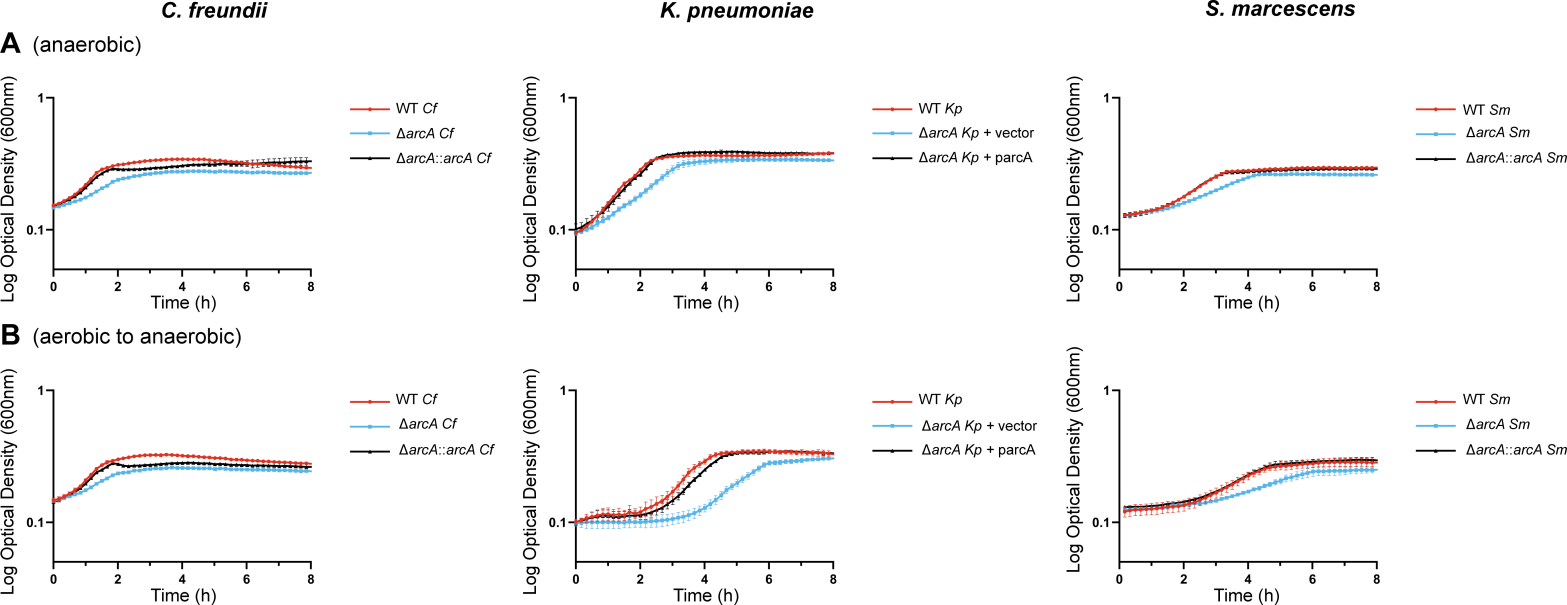
Growth defects of the *K. pneumoniae* and *S. marcescens* Δ*arcA* mutants are more pronounced during the aerobic to anaerobic transition. Wild-type strains and derivatives were cultured overnight in LB under (**A**) anaerobic or (**B**) aerobic conditions and then normalized based on OD_600_. Fresh LB was inoculated with normalized overnight cultures in an anaerobic chamber. OD_600_ was then measured with a plate reader every 10 min. The graphs presented here are representative of three independent experiments. Each culture was grown in triplicate, and the average with standard deviation was plotted over time.

In model species, the ArcB kinase is a sensor of anaerobiosis and activates ArcA via phosphorylation under such conditions. *arcA* mutant cells were thus hypothesized to exhibit growth defects in the absence of oxygen. Wild-type strains, *arcA* mutant constructs, and genetically complemented constructs or revertants were cultured anaerobically to study how ArcA influences bacterial replication across species (Fig. 3A). The difference in generation times between wild-type and *arcA* mutant constructs was significant for *C. freundii* (73.5 vs 127.1 min) and *S. marcescens* (113.0 vs 173.0 min) but more modest for *K. pneumoniae* (59.6 vs 90.0 min) (Table 2). As opposed to simply responding to anaerobic conditions, ArcB more accurately senses a decrease in oxygen consumption within the cell (21). To induce a reduction in oxygen utilization, cells were cultured aerobically overnight and transferred to a strict anaerobic environment before sub-culturing (Fig. 3B). Shifted growth curves from this condition revealed a more substantial delay in the growth of the *K. pneumoniae* and *S. marcescens arcA* mutants compared to the wild-type strains. The *C. freundii*, *K. pneumoniae,* and *S. marcescens arcA* mutant constructs had 57.5, 22.0, and 72.3 min longer doubling time relative to the respective wild-type strains after transition from aerobic to anaerobic conditions (Table 2). The average doubling time following this transition was very similar to the strict anaerobic condition for the *C. freundii* and *K. pneumoniae* groups. These values were considerably longer for *S. marcescens* cells, but the wild-type strain continued to grow faster than the *arcA* mutant construct. Differences in lag time, or the time to reach maximum growth rate, were also calculated (Δ_LT_) as a metric of the ability of the cells to optimize growth performance (Table 3). The Δ_LT_ values for *C. freundii* and *K. pneumoniae* were greater in the anaerobic condition, indicating the *arcA* mutant took longer to reach its maximum growth rate relative to the wild-type strain. In contrast, the Δ_LT_ was 29.4 min longer in the aerobic to anaerobic transition between the *S. marcescens* wild-type strain and *arcA* mutant construct in comparison to the anaerobic condition.

**TABLE 2:** Doubling times in LB medium (min)

Genotype	Anaerobic	Aerobic → anaerobic
***C. freundii***		
WT	73.5 ± 5.7	72.0 ± 0.7
Δ*arcA*	127.1 ± 7.3	129.5 ± 9.8
Δ*arcA*::*arcA*	80.8 ± 1.2	80.3 ± 2.8
***K. pneumoniae***		
WT	59.6 ± 2.9	65.1 ± 4.0
Δ*arcA +* pBBR1MCS-5	90.0 ± 4.6	87.0 ± 3.9
Δ*arcA +* pBBR1MCS-5+*arcA*	67.8 ± 5.9	73.2 ± 6.8
***S. marcescens***		
WT	113.0 ± 2.7	135.6 ± 16.2
Δ*arcA*	173.0 ± 7.7	207.9 ± 34.0
Δ*arcA*::*arcA*	111.0 ± 6.9	134.7 ± 4.4

**TABLE 3:** Difference in lag times (wild-type strain vs *arcA* mutants) in LB medium (min)

Species	Anaerobic	Aerobic → anaerobic
*C. freundii*	26.8 ± 5.8	10.0 ± 8.2
*K. pneumoniae*	83.6 ± 8.2	70.2 ± 8.2
*S. marcescens*	36.8 ± 20.9	60.2 ± 25.9

Replication of *arcA* mutants was also measured in M9 medium supplemented with glucose and casamino acids to determine if a carbohydrate carbon source alters *arcA*-dependance. The *C. freundii arcA* mutant exhibited a severe growth defect in the presence of glucose for anaerobic culture and aerobic to anaerobic transition culture (Fig. S3), for which both phenotypes were more pronounced than in LB medium (Fig. 3). Growth defects of the *K. pneumoniae arcA* mutant on the other hand were very similar in glucose-containing medium (Fig. S3) to those observed in LB (Fig. 3). In the presence of glucose, all three *S*. *marcescens* cultures displayed a biphasic growth pattern, with the *arcA* mutant displaying the largest growth defect when bacteria were shifted from aerobic to anaerobic conditions (Fig. S3). Overall, the presence of glucose as an available carbon source did not diminish the overall contribution of *arcA* in these three species and indeed exacerbated *arcA*-dependent replication defects for *C. freundii* and *S. marcescens*. The *in vitro* growth kinetics of *arcA* mutants determined here may in part provide a basis for the observed competitive disadvantage of *arcA* mutants during infection, considering that both peptide and monosaccharide carbon sources are likely abundant in the host. Furthermore, limited oxygen availability during infection likely plays an important role in how ArcA modulates metabolism of these three species in the bloodstream and tissue environments. Given the complexity of the infection environment, the potential for ArcA to integrate other relevant signals was investigated.

### Growth in iron-limited medium

**Fig 4:**
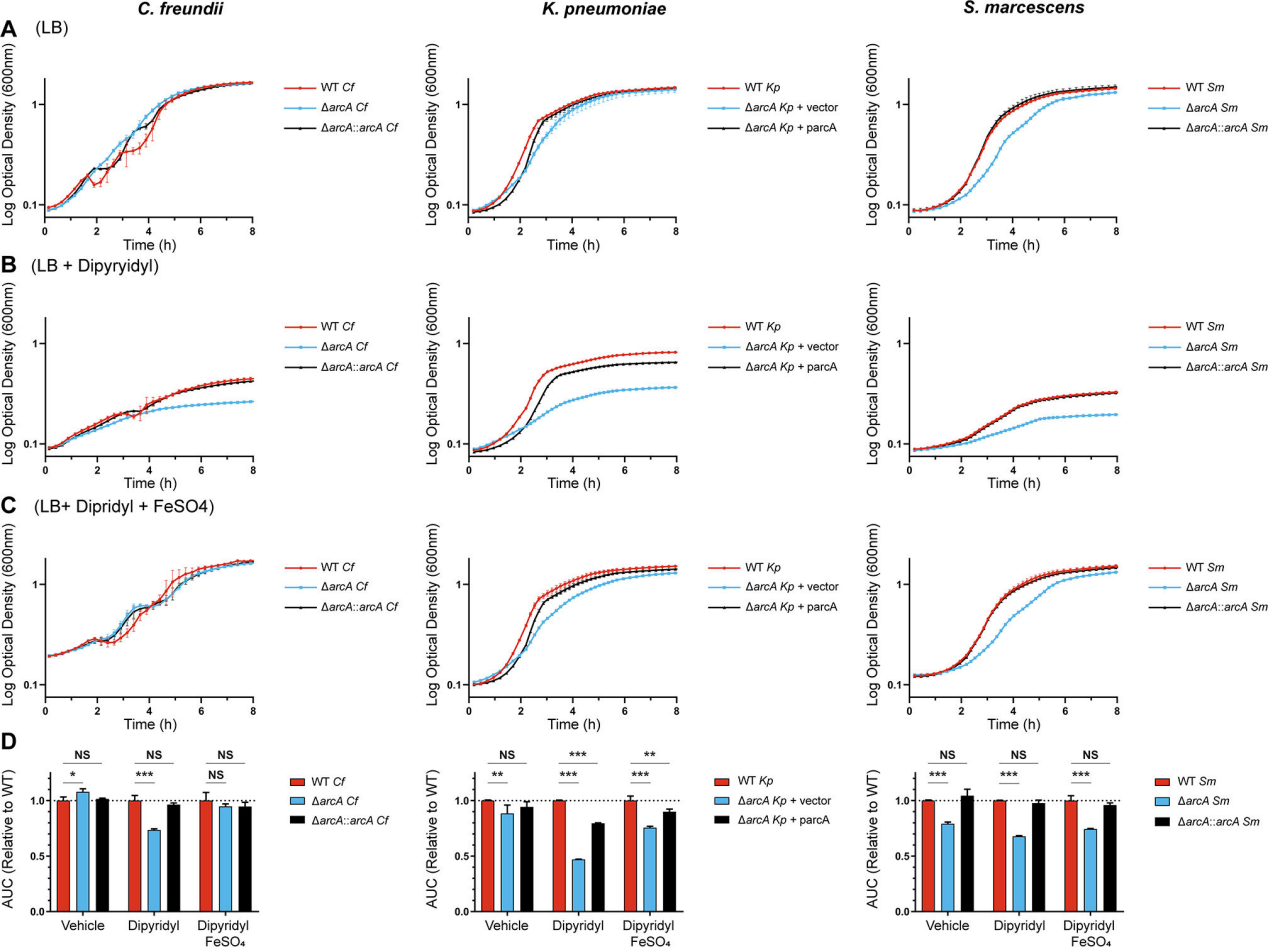
ArcA optimizes growth in an iron-limited medium under aerobic conditions. Overnight cultures incubated aerobically in LB were inoculated into fresh LB containing (**A**) DMSO, (**B**) dipyridyl, or (**C**) dipyridyl supplemented with FeSO_4_. Cultures were incubated at 37°C in aerobic conditions and growth was tracked via OD_600_ by a plate reader every 15 min. Growth curves are the average of technical triplicates with standard deviation and are representative of three independent experiments. (**D**) Growth was assessed by calculating area under the curve (AUC) and comparing this value to the AUC of the wild-type in each condition. Bars represent the average of the technical triplicates of the representative growth curves with standard deviation. Significance was determined by comparing the wild-type strain with the mutant and complemented/reversion constructs with Dunnett’s multiple comparisons test. *P*-values: *≤0.05, **≤0.01, ***≤0.001, NS = not significant.

Iron is a critical cofactor for many respiratory enzymes including succinate dehydrogenase and NADH:ubiquinone oxidoreductase. These electron transport chain (ETC) complexes require iron-sulfur clusters (43) and are transcriptionally repressed by ArcA (18, 19). We hypothesized ArcA would reprogram metabolism during iron limitation to suppress respiratory activity. This regulation would be important in the host where most iron is bound to hemoglobin and sequestered away from invading pathogens by iron-chelating proteins such as ferritin and transferrin (44, 45). Compared to untreated cultures (Fig. 4A), *arcA* mutants grew more slowly than isogenic wild-type strains when cultured aerobically in LB supplemented with the non-utilizable iron chelator 2–2′-dipyridyl (Fig. 4B). Density at stationary phase was considerably lower in the *arcA* mutant cultures than wild-type cultures, which differs from the results of the previous anaerobic experiments where mutant cultures routinely reached the density of the wild-type cells despite any slower growth rates or extended lag periods. The phenotype also demonstrates a requirement for ArcA in the presence of oxygen. In all cases, growth kinetics of the three tested species returned to untreated conditions following supplementation of excess iron to dipyridyl-containing cultures (Fig. 4C). The requirement for ArcA in iron-limited environments is further supported by measuring total growth potential of each species via area under the curve (Fig. 4D). These results ultimately provide an example in which ArcA contributes to growth optimization in response to host-mediated micronutrient limitation.

### Sensitivity to human serum

**Fig 5:**
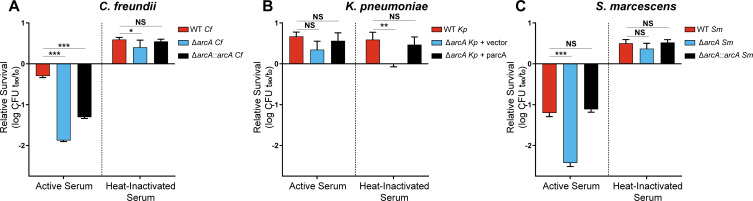
ArcA is required for serum resistance of *C. freundii* and *S. marcescens*. Overnight cultures incubated in LB medium were sub-cultured into LB medium and incubated aerobically until mid-log phase. Cells were normalized and resuspended in normal and heat-inactivated human serum to a final concentration of approximately 2 × 10^8^ CFU/mL. Cultures were then incubated at 37°C for 90 min with sampling before and after incubation for CFU enumeration. Each species was treated with an empirically determined concentration of human serum at the following final concentrations: (**A**) *C. freundii*: 10%; (**B**) *K. pneumoniae*: 90%; (**C**) *S. marcescens*: 40%. Average values of technical triplicates with standard deviation are presented on each graph and are representative of three independent experiments. Significance was determined by comparing the wild-type strain with the mutant and complemented/reversion constructs with Dunnett’s multiple comparisons test. *P*-values: *≤0.05, **≤0.01, ***≤0.001, NS = not significant.

The cell envelope provides the structural barrier necessary to maintain proton motive force (PMF) from the electron transport chain during respiration. Through quinones, the electron transport chain impacts the kinase activity of ArcB (46, 47). ArcA regulates genes whose products maintain the cell envelope in coordination with other regulators such as σ_E_ (19, 48 – 50). The bactericidal effects of serum largely target the bacterial envelope (51), and we therefore investigated the role of ArcA in resisting this infection-relevant envelope stress. The viability of wild-type and *arcA* mutants was quantified in the presence of pooled normal and heat-inactivated human serum. The *C. freundii arcA* mutant was 37.7-fold more susceptible to killing by intact serum relative to the wild-type strain (Fig. 5A). This phenotype was partially rescued in the Δ*arcA::arcA* revertant. In contrast, the *C. freundii* wild-type strain and derivatives grew in culture with heat-inactivated serum, but the *arcA* mutant did not grow as robustly. Neither the *K. pneumoniae* wild-type strain nor the mutant construct exhibited reduced viability when cultured with 90% human serum, which demonstrated a high level of serum resistance for this species (Fig. 5B). The wild-type and complemented *arcA* strain *K. pneumoniae* grew to similar levels in heat-inactivated serum while the *arcA* mutant showed a significantly reduced ability to replicate in serum. This observation was unexpected and may be due to disruption of heat-labile nutrients that *K. pneumoniae* utilizes in an ArcA-dependent manner. Serum-mediated cell death was observed in the *S. marcescens* cultures where the *arcA* mutant experienced 16.7 times more killing relative to the wild-type strain (Fig. 5C). The *S. marcescens* wild-type strain and derivatives cultured in the heat-inactivated serum experienced net growth rather than killing to similar levels as the *C. freundii* group. Disparities in growth between mutant and wild-type strains in heat-inactivated serum suggests that nutrient limitation or another serum-specific growth condition likely contributes to these results. Nevertheless, ArcA influences complement resistance for *C. freundii* and *S. marcescens*, demonstrating the connection of this response regulator to membrane integrity.

### Response to polymyxin B

**Fig 6:**
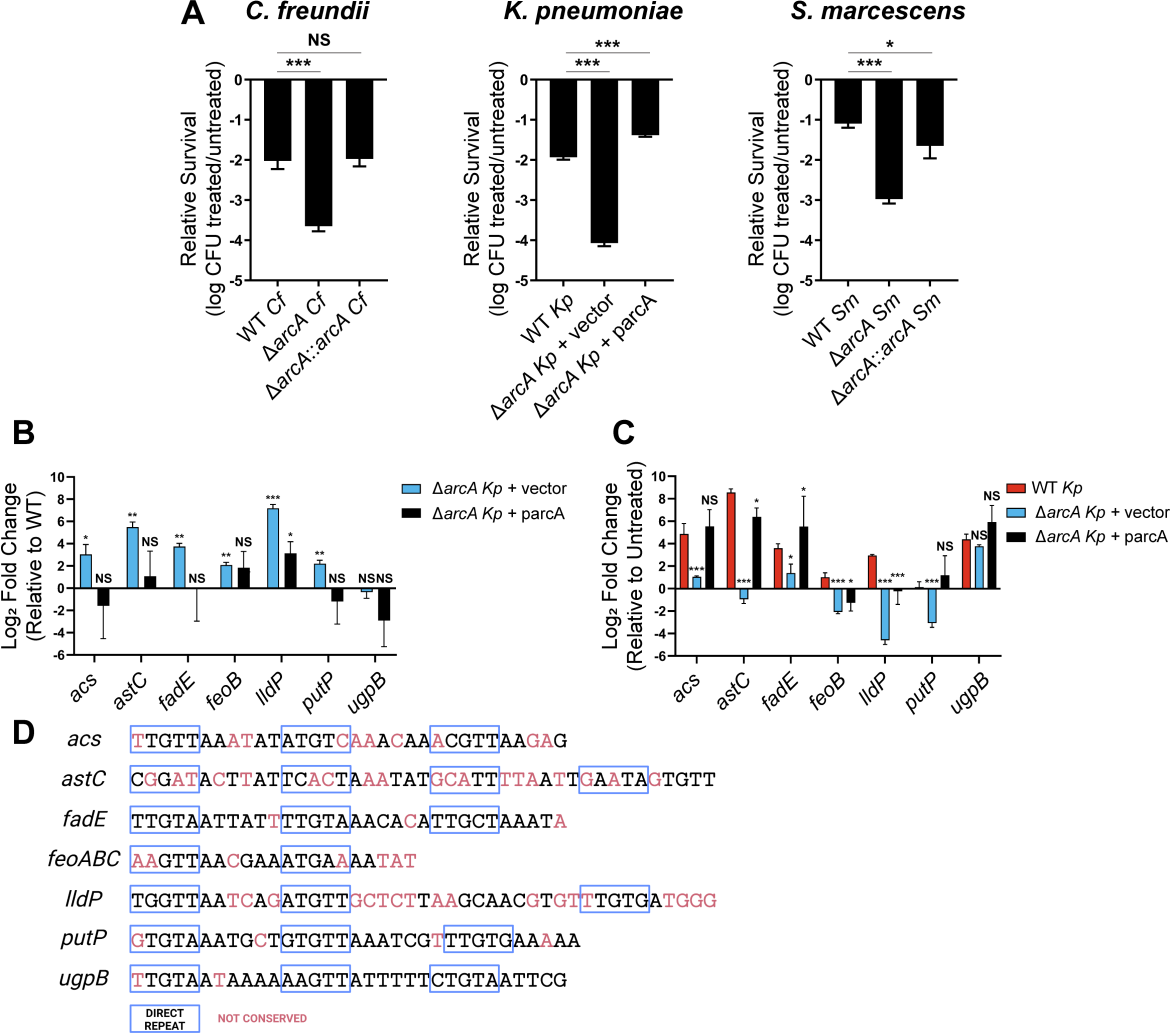
ArcA is involved in the polymyxin B response. (**A**) Overnight cultures grown in LB medium were sub-cultured into LB medium and incubated aerobically to mid-log phase. Cultures were normalized to an OD_600_ 0.2 and treated with polymyxin B for 1 h at 37°C. Survival was assessed relative to untreated cultures, and the log transformed data are presented as an average of technical triplicates. Each graph is representative of three independent experiments. Significance was determined by comparing the wild-type strain with the mutant and complemented constructs with Dunnett’s multiple comparisons test. (**B and C**) To measure expression of candidate ArcA-regulated genes in the *K. pneumoniae* wild-type strain and derivatives, mid-log phase cells grown in LB were normalized to approximately 2 × 10^8^ CFU/mL in PBS. Cells were treated with 5 µg/mL polymyxin B for 15 min followed by RNA extraction. RT-qPCR was performed to assess expression of *acs, astC, fadE, feoB, lldP, putP,* and *ugpB* with *gap* serving as the housekeeping gene. Results are displayed as log_2_ fold change and are the average of three biological replicates with standard deviation. (**B**) In untreated conditions, expression of each gene by the mutant and complemented strains was compared to that of the wild-type strain following normalizing of Ct values to *gap* and log transformation. Significance was determined via a one-sample *t*-test with a null hypothetical value of zero. (**C**) Expression of each gene was then compared between untreated and polymyxin B conditions for each genotype. Significance was determined by comparing the wild-type strain with the mutant and complemented constructs with Dunnett’s multiple comparisons test. (**D**) FIMO (55) was utilized to search for ArcA-binding boxes from *E. coli* K-12 MG1255 (18) in the promoter regions of the seven genes evaluated in the expression studies. Sequences that had a *P-*value and *Q-*value at or below 0.05 were considered significant. In the promoters of 6/7 *K*. *pneumoniae* genes, a putative ArcA-binding sequence was identified. Underlined, red nucleotides were loci not conserved between *E. coli* and *K. pneumoniae* sequences. Direct repeats within sequences were labeled based on coordinates of direct repeats within corresponding promoters of *E. coli* genes and are denoted by blue boxes. *P*-values: *≤0.05, **≤0.01, ***≤0.001, NS = not significant.

The host innate immune response includes cationic antimicrobial peptides (CAMPs) which permeabilize Gram-negative bacterial cell membranes (52). The model CAMP polymyxin B (PMB) was used to test if ArcA responds to CAMP-mediated cell membrane damage (53, 54). PMB treatment of mid-exponential phase cells demonstrated that *arcA* mutants of all three species were significantly more susceptible to killing than isogenic wild-type strains and complemented and revertant constructs (Fig. 6A). Survival rates were 44-, 138-, and 76-fold higher in the wild-type strains relative to the *arcA* mutant constructs of *C. freundii*, *K. pneumoniae*, and *S. marcescens*, respectively. These results are especially notable for *K. pneumoniae* given the lack of *arcA*-dependent serum resistance (Fig. 5B) and imply ArcA also responds to *K. pneumoniae* membrane perturbation. Together, these data support previous findings that ArcA regulation is important for cellular processes supporting envelope health. To investigate further, an ArcA-specific genetic response to PMB was interrogated.

The genes differentially expressed by *K. pneumoniae* in response to polymyxin B have been previously identified via RNA-seq (56), and we hypothesized that a subset of these genes is regulated by ArcA. To explore this, the *K. pneumoniae* data set was compared to the genes and operons directly controlled by ArcA in *E. coli* under anaerobic conditions (18). A list was generated of candidate genetic elements controlled by ArcA in response to PMB. After removal of genes with less than 80% shared amino acid identity between *E. coli* CFT073 and *K. pneumoniae* KPPR1, *acs*, *astC*, *fadE, feoB, lldP, putP,* and *ugpB* were selected for expression studies. qRT-PCR was used to measure gene expression of these targets in mid-exponential *K. pneumoniae* cells following sub-lethal treatment with PMB. In untreated control conditions, every gene except *ugpB* was more highly expressed in the *arcA* mutant relative to the wild-type strain (Fig. 6B). This result matches *E. coli* studies in which ArcA serves as a repressor for all of these genes except *feoB* under strict anaerobic conditions (18, 19). In all cases, genetic complementation reduced transcript levels compared to the *arcA* mutant. In the PMB treatment condition, *acs*, *astC*, *fadE, feoB, lldP*, and *ugpB* were upregulated 2.0-fold to more than 375-fold relative to untreated conditions in the wild-type cells (Fig. 6C). *putP* exhibited minimal induction in response to PMB. The complemented construct yielded largely similar results to the wild-type strain excluding *feoB* and *lldP* for which intermediate phenotypes were noted. *acs* (1.9-fold) and *fadE* (6.4-fold) were also upregulated in *arcA* mutant cells, but these levels were significantly lower compared to wild-type. In contrast to wild-type, the following genes were downregulated in the *arcA* mutant following polymyxin B exposure: *astC* (2.6-fold), *feoB* (4.7-fold), *lldP* (24.9-fold), and *putP* (9.8-fold). In summary, six out of the seven genes were suppressed by ArcA in untreated conditions while ArcA served as an activator or mediator of de-repression of the same genes in response to PMB-induced stress.

Promoters of the *K. pneumoniae* PMB-induced transcripts were analyzed to search for ArcA-binding sites based on previously reported *E. coli* sites (18). A motif identification tool was used to identify putative-binding sites in the homologous promoter regions of *K. pneumoniae*. Potential ArcA-binding sequences were identified for all *K. pneumoniae* genes including *ugpB* which was not differentially expressed in an ArcA-dependent manner in response to PMB (Fig. 6D). Coordinates of the direct repeats bound by ArcA in the *E. coli* sequences were mapped onto the *K. pneumoniae* sequences. Most of the nucleotide differences between the *E. coli* and *K. pneumoniae* sequences were outside of the direct repeats, suggesting a pressure for conservation of these motifs. Putative ArcA-binding sites were also readily identifiable for many of the same *C. freundii* and *S. marcescens* genes (Fig. S4). The PMB survival assay, expression data, and putative ArcA-binding sites all provide evidence for a direct role of ArcA in responding to CAMPs, further highlighting its function during infection.

### Fermentation following electron transport chain perturbations

**Fig 7:**
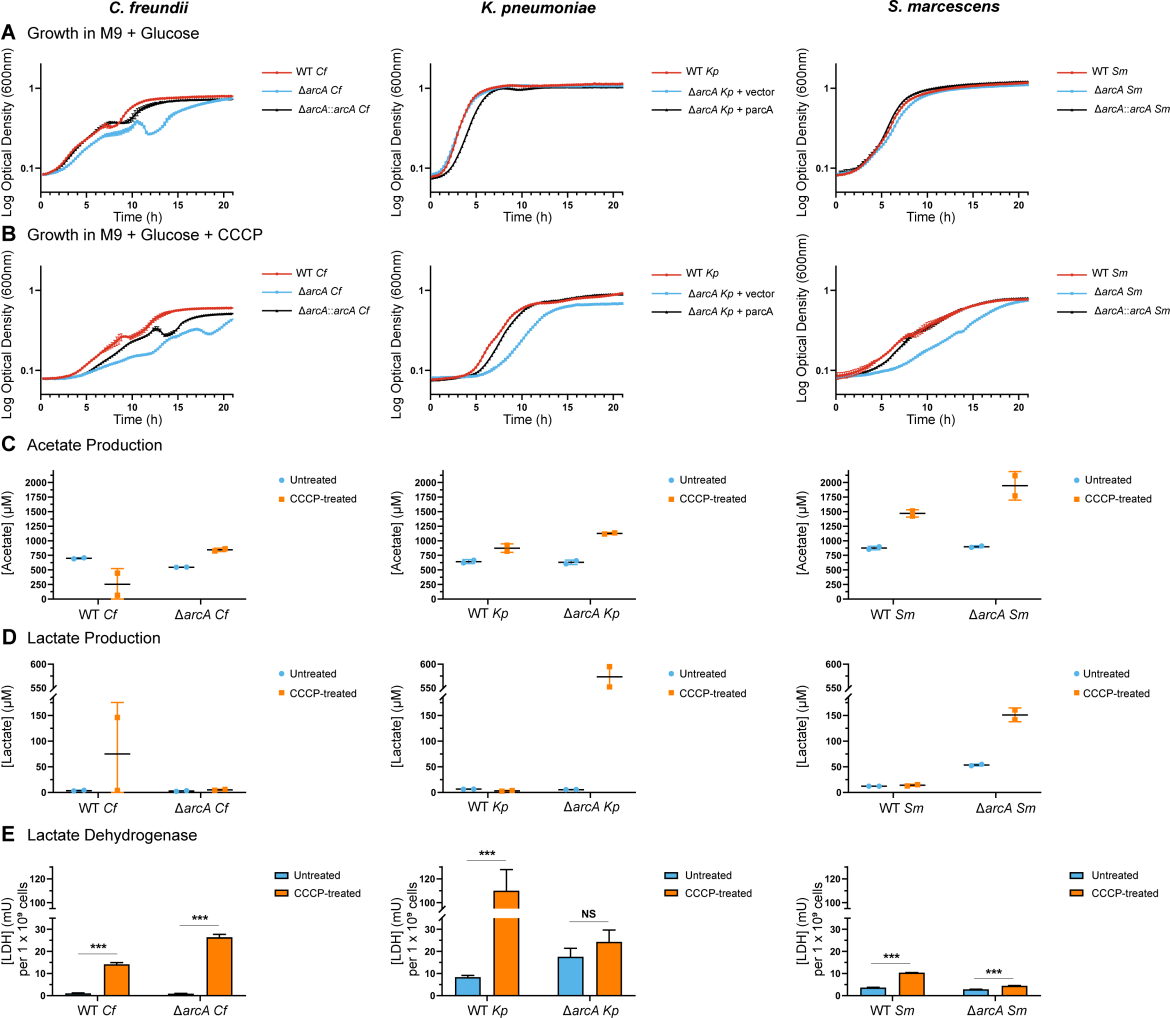
ArcA modulates metabolism in response to disruption of proton motive force by the uncoupler carbonylcyanide-m-chlorophenylhydrazone (CCCP). The ability of wild-type and Δ*arcA* mutant cells to respond to disruption of ATP synthesis via oxidative phosphorylation despite the availability of glucose and oxygen was tested. Overnight cultures incubated aerobically in LB were inoculated into M9 minimal medium with 0.4% glucose without (**A**) or with (**B**) CCCP (*C. freundii*, 15 µM CCCP; *K. pneumoniae*, 20 µM CCCP; *S. marcescens*, 25 µM CCCP). Cultures were incubated at 37°C under aerobic conditions and growth was tracked via OD_600_ by a plate reader every 15 min. Growth curves are the average of technical triplicates with standard deviation and are representative of three independent experiments. (**C**) Targeted metabolomics by LC-MS was utilized to quantitate acetate from supernatants of wild-type and *arcA* mutant cultures in early exponential phase from the same conditions as the growth curves. The averages of two biological samples with standard deviation are presented in each graph. (**D**) d-Lactate dehydrogenase (d-LDH) was measured from cell lysates of cultures grown in M9 minimal medium with 0.4% glucose without or with CCCP at the same concentrations as the growth curve conditions. d-LDH levels were quantified with Amplite Fluorimetric D-Lactate Dehydrogenase Assay Kit (AAT Bioquest) by comparing sample readings to known standards. d-LDH levels were normalized per 1 × 10^9^ cells. The average of three technical replicates with standard deviation is presented as representative of three independent experiments. LDH levels were compared between cells in untreated and treated conditions using Šídák’s multiple comparisons test to determine significance. (**E**) Targeted metabolomics was repeated to quantify lactate with the same experimental set-up as acetate (**C**). See Materials and Methods for details, Fig. S5 for sampling metrics, and Fig. S6 for LC-MS acetate and lactate samples. *P*-values: *≤0.05, **≤0.01, ***≤0.001, NS = not significant.

Cell envelope damage can compromise maintenance of a proton gradient across the inner membrane by the ETC. When a PMF cannot be maintained, ATP production via chemiosmosis is not possible, and cells must rely on metabolic pathways independent of the ETC for energy production. The ability to switch to such processes following membrane damage likely requires metabolic regulators such as ArcA to repress respiratory complexes and pathways feeding the ETC (18, 57). Carbonylcyanide-*m*-chlorophenylhydrazone (CCCP) is a PMF uncoupler and was utilized to test the role of ArcA in optimizing growth following inhibition of aerobic respiration. Cells were cultured aerobically in a minimal medium containing glucose with and without CCCP to test the hypothesis that ArcA activity supports growth when chemiosmosis is not possible despite the availability of electron donors and a terminal electron acceptor. The wild-type and *arcA* mutants of *K. pneumoniae and S. marcescens* grew nearly identically to their isogenic wild-type strains in an untreated minimal medium supplemented with glucose, whereas the *C. freundii arcA* mutant experienced a relatively minor lag (Fig. 7A). Following CCCP treatment, *C. freundii*, *K. pneumoniae*, and *S. marcescens arcA* mutants had longer lag times of 8.0 h, 4.2 h, and 8.3 h relative to wild-type strains, respectively (Fig. 7B). These delays in growth are mirrored by longer doubling times of the *C. freundii* (25.2 min), *K. pneumoniae* (32.1 min), and *S. marcescens* (18.7 min) *arcA* mutants relative to the wild-type strains. The growth defects overall support a role of ArcA in optimizing growth in the presence of CCCP for all three species.

Growth in CCCP theoretically requires an ETC-independent mechanism for ATP production, such as fermentation. ArcA mediates the transition to fermentation, and *E. coli arcA* mutants excrete a different profile of fermentative products as compared to wild-type cells under microaerobic and anaerobic conditions (58 – 60). The *arcA* mutant bacteria described in this study were hypothesized to experience the same defects in mixed acid fermentative processes following PMF uncoupling (61 – 64). Acetate and lactate were quantified by high-performance liquid chromatography (HPLC) in the supernatant of untreated and CCCP-treated cultures as metrics of fermentation (Fig. S5 and 6). Acetate levels decreased in wild-type *C. freundii* 6.2-fold but were 1.5 times higher in the *arcA* mutant relative to untreated conditions (Fig. 7C). In the *K. pneumoniae* and *S. marcescens* wild-type strains and *arcA* mutants, acetate levels were 1.4 to 2.2-fold higher in CCCP-treated conditions (Fig. 7C). Lactate increased 20.7-fold in the wild-type *C. freundii* in CCCP but did not change in the *arcA* mutant (Fig. 7D). Almost no differences in excreted lactate were observed between the untreated and CCCP cultures of wild-type *K. pneumoniae* and *S. marcescens* whereas lactate levels increased in the respective *arcA* mutants by 102.0-fold and 2.8-fold (Fig. 7D). In *E. coli arcA* mutant cultures, supernatant acetate levels stay the same or decrease whereas lactate levels increase (59, 60, 65). Fermentation induced by CCCP or an absence of oxygen may favor production of different fermentative end-products, and ArcA-mediated fermentation is likely species-specific.

Targeted metabolomics revealed a different fermentative profile following CCCP treatment of wild-type and *arcA* strains, but conclusions from these studies are limited by the potential recycling of secondary metabolites by cells. D-lactate dehydrogenase levels (LDH) were thus measured as an additional metric of fermentation to continuing testing the hypothesis that *arcA* mutants exhibit a dysregulated response to CCCP treatment. LDH activity significantly increased in the *C. freundii* wild-type strain (13.5-fold) and *arcA* mutant construct (30.3-fold) cultured with CCCP relative to untreated conditions, indicating fermentation was induced but that ArcA may play an inhibitory role of LDH (Fig. 7E). Relative LDH levels also increased in wild-type *K. pneumoniae* (13.2-fold) and *S. marcescens* (2.8-fold) CCCP cultures, and LDH increases were ArcA-dependent for *K. pneumoniae* and partially so for *S. marcescens* (Fig. 7E). The inverse correlation of higher LDH levels in wild-type cells to lower lactate concentrations in their supernatant (Fig. 7D) is not clear but might be explained by unknown effects of CCCP or lactate oxidation at the ETC (66). Nevertheless, differences in LDH levels between wild-type and *arcA* mutant cultures provides further evidence that ArcA plays a role in the transition to fermentation following uncoupling of PMF.

## DISCUSSION

**Fig 8:**
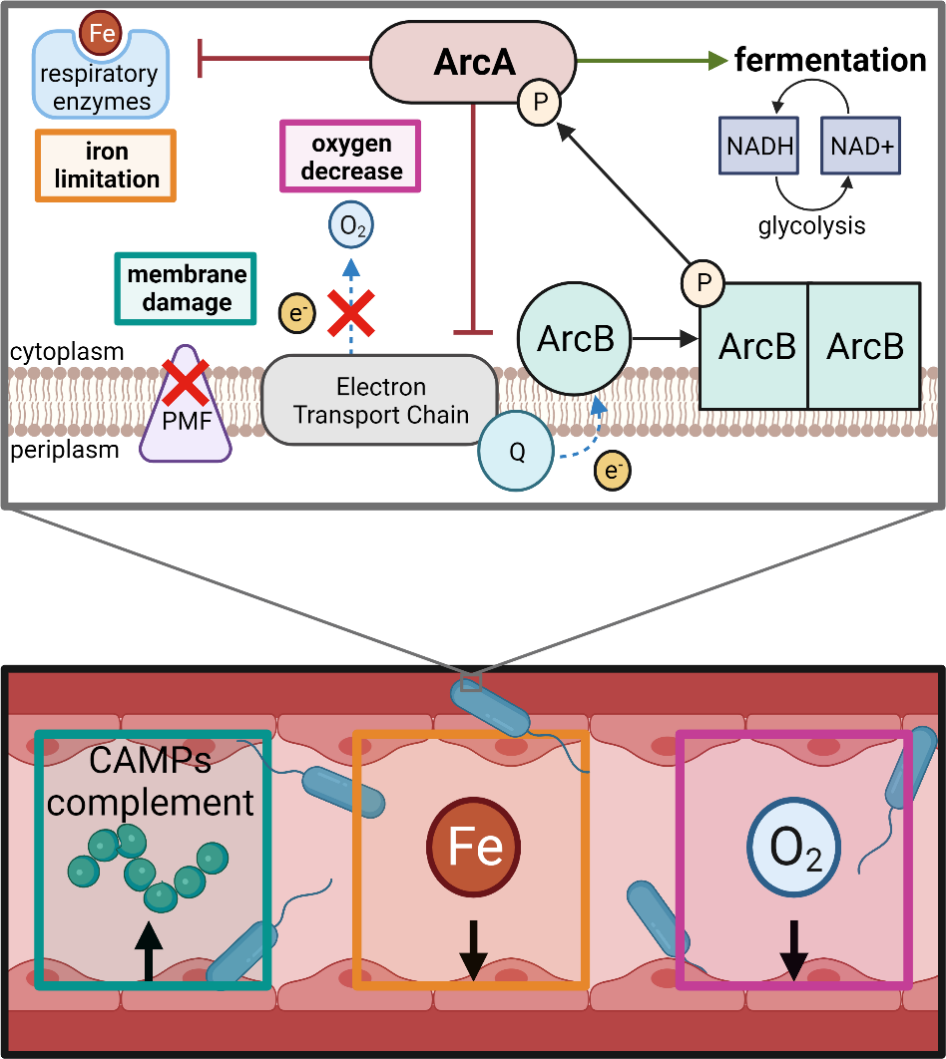
Response regulator ArcA supports fitness during Gram-negative bacteremia. Within the mammalian bloodstream, bacteria encounter decreased iron (Fe) availability, oxygen (**O_2_**) levels, and elements of the host innate immune response such as cationic antimicrobial peptides (CAMPs) which can cause membrane damage. ArcA mediates the transition to fermentation in response to such conditions unfavorable for respiration including the inability to maintain a proton motive force (PMF). Quinones (**Q**) of the electron transport chain transfer electrons to sensor kinase ArcB instead of to pathways which lead to oxygen as the terminal electron acceptor. ArcB then phosphorylates and activates ArcA in response to decreased electron transport chain activity, providing a mechanism by which ArcA can respond to multiple stimuli impacting metabolic activity within the cell.

The response regulator ArcA is highly conserved among *Enterobacterales* species and was demonstrated for the first time here to promote fitness of *C. freundii*, *K. pneumoniae*, and *S. marcescens* during bacteremia. *arcA* mutants exhibited a dysregulated response to changes in oxygen and iron availability, conditions likely to be encountered during infection. ArcA was found to be part of the response to membrane damage caused by the CAMP polymyxin B, demonstrating an expanded role for ArcA linked to disruption of ETC activity. ArcA mediated a shift to fermentation in response to PMF uncoupling, independent of oxygen availability, as measured by LDH activity. The proposed model detailing how ArcA responds to low oxygen, limited iron, and membrane damage is summarized in Fig. 8.

Bacteria entering the bloodstream directly from the environment or another infection site expectedly encounter increasingly anaerobic conditions during dissemination. Ambient oxygen levels of approximately 21.1% decrease from 13.2% in arterial blood to 5.4% in the liver with only small amounts being dissolved as 98% is hemoglobin-bound (67, 68). The *in vitro* growth defects of the *K. pneumoniae* and *S. marcescens arcA* mutants were evident by a sizeable shift in growth curves in the aerobic to anaerobic transition. Our group has calculated the average population doubling times of *C. freundii* (66 min), *K. pneumoniae* (39 min), and *S. marcescens* (61 min) in murine spleens (38), leading to the conjecture that bacterial cells’ ability to maintain rapid replication rates is an important factor in combating host clearance mechanisms and establishing infection during bacteremia. This study captures the response of ArcA to changes in oxygen utilization and showcases the need for ArcA regulation to maintain such rapid growth. During urinary tract infections, *E. coli* utilizes the TCA cycle while glycolysis is dispensable (69, 70). If *E. coli* favors the same pathways during bacteremia, ArcA would be expendable as a repressor of the TCA cycle, explaining the lack of fitness defect of the *E. coli arcA* mutant during bacteremia. Our previous TnSeq screens identified genes encoding 6-phosphofructokinase, phosphate acetyltransferase, and acetate kinase as contributing to fitness for *C. freundii* and *S. marcescens* during bacteremia, suggesting glycolysis is utilized in infection (10, 11).

ArcA maximizes replication of *C. freundii*, *K. pneumoniae*, and *S. marcescens* in iron-limited conditions and reportedly regulates iron homeostasis alongside FNR and Fur in *E. coli* (71). Stunted growth of *arcA* mutants in iron limitation occurred under aerobic conditions, further showing ArcA responds to decreased oxygen utilization rather the absence of oxygen. Fermentation is the preferred metabolic pathway during iron starvation, and Chareyre et al. demonstrated iron deprivation leads to post-transcriptional repression of respiratory complexes by small RNA RhyB (72, 73). The *nuo* and *shd* operons encoding these complexes are strongly repressed by ArcA (18, 19), indicating coordination between RhyB and ArcA during iron limitation may exist. The link between iron and oxygen is observed in higher order species as human Hypoxia Inducible Factor (HIF), a transcriptional activator induced by low oxygen levels, also promotes glycolytic activity during iron limitation (74 – 76).

These studies are the first to our knowledge linking ArcA to CAMP sensitivity. Upregulation of six genes by ArcA following PMB treatment was unexpected given its well-established role in repressing five of them (18 – 20). Upregulation or downregulation by ArcA of the same gene depending on growth conditions has precedent (77). Future studies can determine if ArcA directly or indirectly upregulates the PMB-responsive genes, which do not encode pathways of aerobic respiration. A “core” ArcA regulon may exist in which ArcA invariably represses central carbon metabolic pathways alongside a “conditional” regulon where its role is contextual. More transcriptomic and DNA footprinting studies will be critical for defining the direct and indirect ArcA regulons in infection-relevant conditions.

CAMPs damage the inner membrane and inhibit respiratory enzymes (78, 79), implying PMB disrupts PMF maintenance or damages the ETC. *arcA* mutants grew more slowly in CCCP, connecting ArcA to ETC perturbations. CCCP induced higher LDH levels in all three species, indicating a shift to fermentation, and this increase was at least partially ArcA-dependent for *K. pneumoniae* and *S. marcescens*. Targeted metabolomics revealed CCCP-induced lactate and acetate production is ArcA and species-dependent. Acetate and lactate pathways contribute to the maintenance redox balance during glycolysis (64). Based on ArcA maintaining intracellular redox balance (14), acetate and lactate production might reflect balancing of redox levels in the presence of CCCP. Cells more efficient in carbon cycling may reuse end products of fermentation rather than secrete them into the supernatant. To this end, an *arcA* mutant of *E. coli* undergoing anaerobic fermentation had a 15.8% lower growth rate relative to the wild-type strain (19). Approaches including carbon tracing and untargeted metabolomics can further characterize the global metabolic changes in these species in response to proton motive force uncoupling.

We conclude that ArcA responds to low oxygen conditions, decreased iron levels, and host-mediated membrane damage during bacteremia in three related Gram-negative bacterial species. Activation of ArcA in response to low iron and membrane damage was not tested, so control of ArcA function in these contexts remains to be established. It remains possible that differences in ArcB activity between species explain some of the ArcA-mediated phenotypes that proved to be differential. Additionally, ArcA has recently been shown to become partially active independently of ArcB under oxidizing conditions, providing evidence of additional regulatory mechanisms that require further study (80). Future ArcA studies will be important in understanding the complex regulation of central carbon pathways utilized in the bloodstream environment and may reveal other shared or unique metabolic capabilities.

## MATERIALS AND METHODS

### Bacterial strains and culture conditions

Bacterial strains and constructs utilized in this study are listed in Table 1. *E. coli* TOP10 cells were used for routine cloning purposes. Overnight culture was performed in LB (81) and experimental cultures were grown in LB or M9 medium (82) containing 100 µM CaCl_2_, 1 mM MgSO_4_, 0.4% D-glucose, and 0.1% casamino acids as indicated. Cultures were maintained at 37°C with 200 RPM shaking unless noted otherwise. Anaerobic cultures were maintained in a 37°C anaerobic chamber maintained at 10% H_2_, 5% CO_2_, and 85% N_2_.

### Strain engineering

*C. freundii, E. coli, K. pneumoniae,* and *S. marcescens arcA* mutants were generated using Lambda red mutagenesis as previously described (10, 83, 84) with the oligonucleotides from Table S1. Chromosomal mutations were confirmed by PCR-amplification and sequencing of the mutant allele. Revertants of the *C. freundii* UMH14 and *S. marcescens* UMH9 Δ*arcA::npII* mutant were generated by re-integration of the wild-type *arcA* allele into the original locus with recombineering. The *K. pneumoniae* KPPR1 *arcA* mutant construct was complemented *in trans* using the pBBR1MCS-5 broad host-range plasmid (85).

### Murine bacteremia model

Overnight LB cultures of wild-type and *arcA* mutant constructs were sub-cultured into fresh LB and cultured at 37°C with 200 RPM shaking. Mid-log cells were washed and resuspended with PBS and normalized by OD_600_ to approximate CFU/mL of 1 × 10^9^ (*C. freundii*)*,* 2 × 10^7^ (*E. coli*), 1 × 10^6^ (*K. pneumoniae*), and 1 × 10^8^ (*S. marcescens*). Wild-type and *arcA* mutant cells were injected into 6–8 wk old male and female C57BL/6 mice (Jackson Laboratory) via tail-veins as previously described (86). Inocula and organ homogenates were plated on LB agar with and without kanamycin (50 µg/mL) for differential CFU determinations. Competitive indices were calculated by dividing the ratio of mutant to wild-type CFU in organs by the inocula ratio. Competitive indices were log-transformed, and significance was determined by a one-sample *t*-test with a hypothetical null value of zero. Murine experiments were performed in compliance with an animal protocol (PRO00010856) approved by the University of Michigan Institutional Animal Care & Use Committee.

### *In vitro* growth

Aerobic and anaerobic overnight cultures were normalized by OD_600_, washed and resuspended in PBS, and sub-cultured 1:100 into the desired media. For aerobic growth studies, 300 µL from each culture was added in triplicate to a honeycomb plate. Iron-limited M9 media with and without iron supplementation were prepared as follows: *C. freundii*—0.6 mM 2,2'-dipyridyl (0.6% DMSO) and 3.0 mM FeSO_4_; *K. pneumoniae*—0.2 mM 2,2'-dipyridyl (0.2% DMSO) and 0.1 mM FeSO_4_; *S. marcescens*—0.4 mM 2,2'-dipyridyl (0.4% DMSO) and 0.2 mM FeSO_4_. Growth was assessed by comparing area under the curve to wild-type strains with significance determined by Dunnett’s multiple comparisons test. M9 media were prepared with carbonylcyanide-*m*-chlorophenylhydrazone (CCCP) at 15 µM (*C. freundii*), 20 µM (*K. pneumoniae*), and 25 µM (*S. marcescens*). Plates were incubated on a Bioscreen-C plate reader with the following settings: 37°C, intermediate continuous shaking, OD_600_ measurement every 15 min. For anaerobic growth studies, 200 µL from each prepared culture was added in triplicate to a 96-well plate, which was incubated in an anaerobic plate reader (BioTek Powerwave HT) per the following settings: 37°C, static, OD_600_ measurement taken every 10 min.

### Survival assays

Pooled human complement serum (Innovative Research) stored at −80°C was thawed directly prior to use and heat-inactivated at 56°C for 45 min, where indicated. Washed mid-log cells were resuspended to a final density of 2 × 10^8^ CFU/mL in PBS and added to serum in 96-well plates. Serum sensitivity was tested at concentrations of 10% (*C. freundii*), 90% (*K. pneumoniae*), or 20% (*S. marcescens*). Bacterial viability was determined after a static 90-min exposure at 37°C by CFU enumeration relative to time zero. For polymyxin B studies, cells were collected by centrifugation and resuspended in PBS to an OD_600_ of 0.2. polymyxin B (RPI) was added to cells in 96-well plates at final concentrations of 5.0 µg/mL (*C. freundii*), 50 µg/mL (*K. pneumoniae*), or 100 µg/mL (*S. marcescens*). Plates were incubated statically for 1 h at 37°C followed by enumeration of viable bacteria relative to untreated conditions. For both assays, Dunnett’s multiple comparisons test was used to assess statistical significance following log transformation of data.

### Gene expression

Mid-exponential phase aerobic bacteria were normalized to 2 × 10^8^ CFU/mL in PBS. Ten microliters of resuspended culture were added to a 125-mL flask, and 1.0 mL of resuspended culture was kept as an untreated control. Polymyxin B (50 µL) was added for a final concentration of 5 µg/mL. Flasks were incubated at 37°C and 200 RPM shaking for 15 min. RT-qPCR was performed with Power SYBR Green (Thermo Fischer) followed by calculation of relative gene expression with the 2^−ΔΔCt^ (Livak) method (87).

### Metabolomics

Supernatant acetate and lactate levels from the CCCP growth curve experimental set-up condition were quantified by the University of Michigan Metabolomics Core via HPLC (88). D-lactate dehydrogenase activity was measured in midexponential cells from the same conditions with the Amplite Fluorimetric D-Lactate Dehydrogenase Assay Kit (AAT Bioquest) per the manufacturer’s instructions.

See Text S1 for additional details of Materials and Methods.

## SUPPLEMENTAL MATERIAL

File S1 mbio.01448-23-s0001.xlsx Genomes and ArcA ORFs analyzed for conservation modeling.

Figure S1 mbio.01448-23-s0002.tif A majority of ArcA residues are evolutionarily conserved across order *Enterobacterales*.

Figure S2 mbio.01448-23-s0003.tif ArcA is highly conserved at the amino acid level.

Figure S3 mbio.01448-23-s0004.tif Growth of bacterial strains in M9 + glucose + casamino acids in anaerobic conditions.

Figure S4 mbio.01448-23-s0005.tif Putative ArcA-binding sequences of *C. freundii* and *S. marcescens*.

Figure S5 mbio.01448-23-s0006.tif Sampling points for targeted metabolomics.

Figure S6 mbio.01448-23-s0007.tif Acetate and lactate standards for LC-MS.

Supplemental Text mbio.01448-23-s0008.docx Supplemental Materials and Methods and figure legends.

Table S1 mbio.01448-23-s0009.docx Primers.

ASM does not own the copyrights to Supplemental Material that may be linked to, or accessed through, an article. The authors have granted ASM a non-exclusive, world-wide license to publish the Supplemental Material files. Please contact the corresponding author directly for reuse.
