# Supplementary figures and images for: Conserved metabolic regulator ArcA responds to oxygen availability, iron limitation, and cell envelope perturbations during bacteremia
Source: mBio. 2023 Sep 8;14(5):e01448-23. doi: 10.1128/mbio.01448-23 (PMC10653796; doi:10.1128/mbio.01448-23)

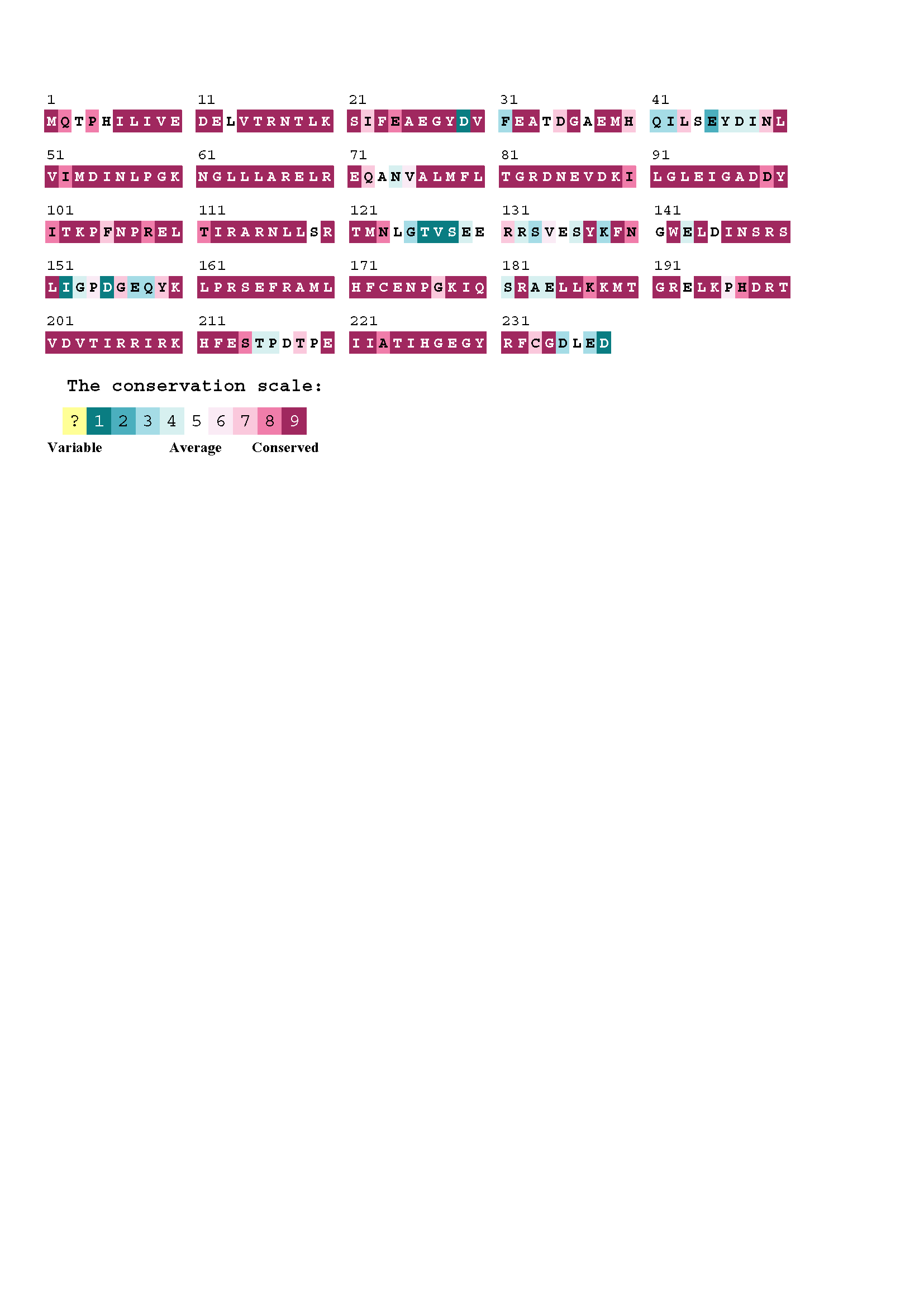

Supplement: Figure S1 — A majority of ArcA residues are evolutionarily conserved across order Enterobacterales. [file mbio.01448-23-s0002.tif]

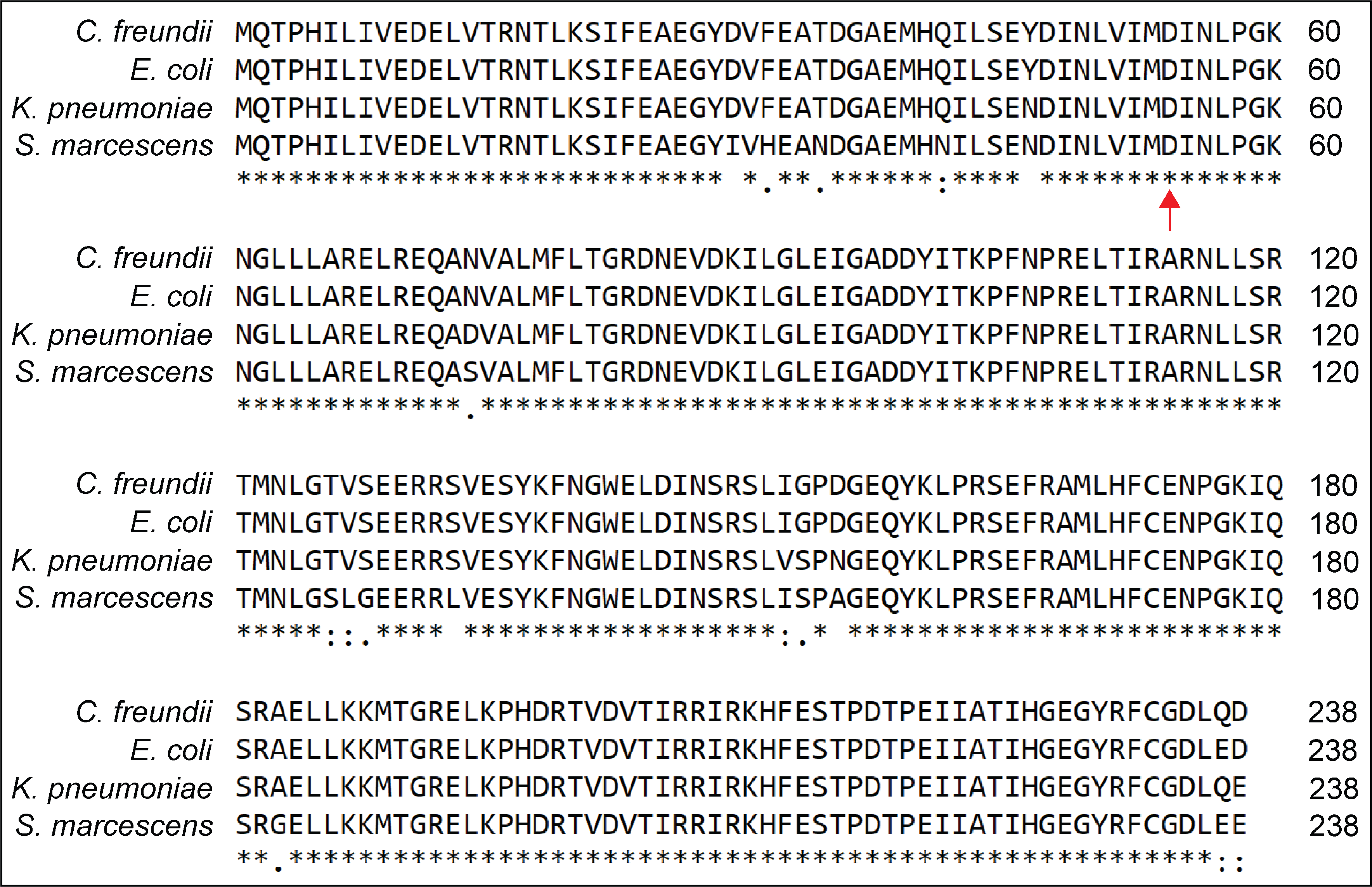

Supplement: Figure S2 — ArcA is highly conserved at the amino acid level. [file mbio.01448-23-s0003.tif]

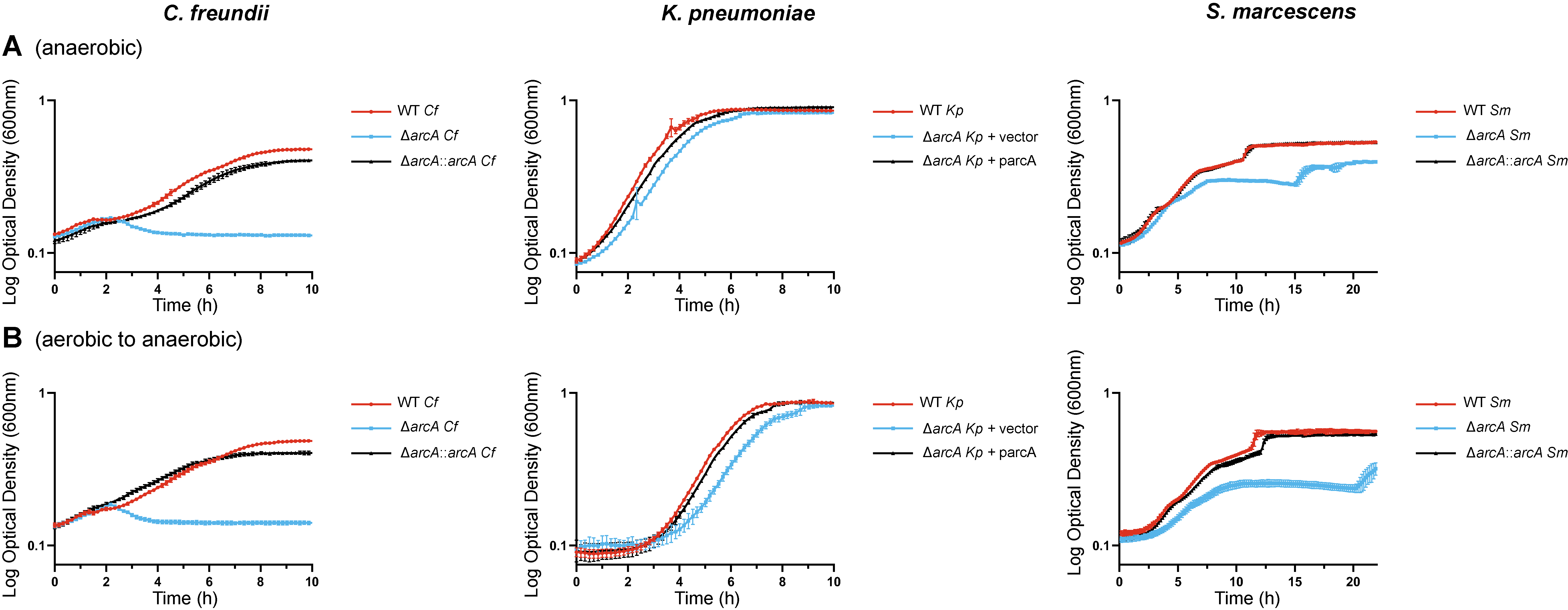

Supplement: Figure S3 — Growth of bacterial strains in M9 + glucose + casamino acids in anaerobic conditions. [file mbio.01448-23-s0004.tif]

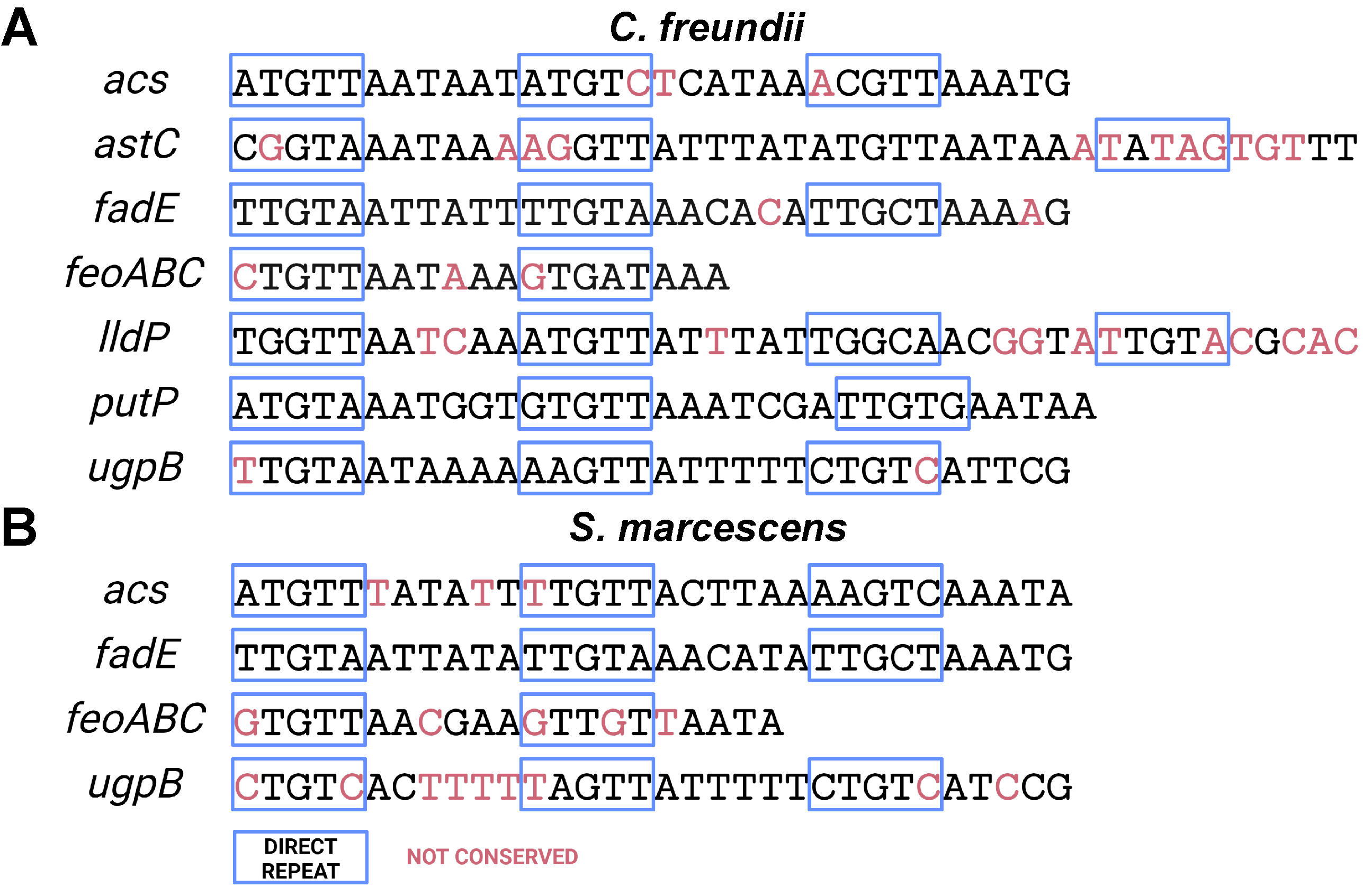

Supplement: Figure S4 — Putative ArcA-binding sequences of C. freundii and S. marcescens. [file mbio.01448-23-s0005.tif]

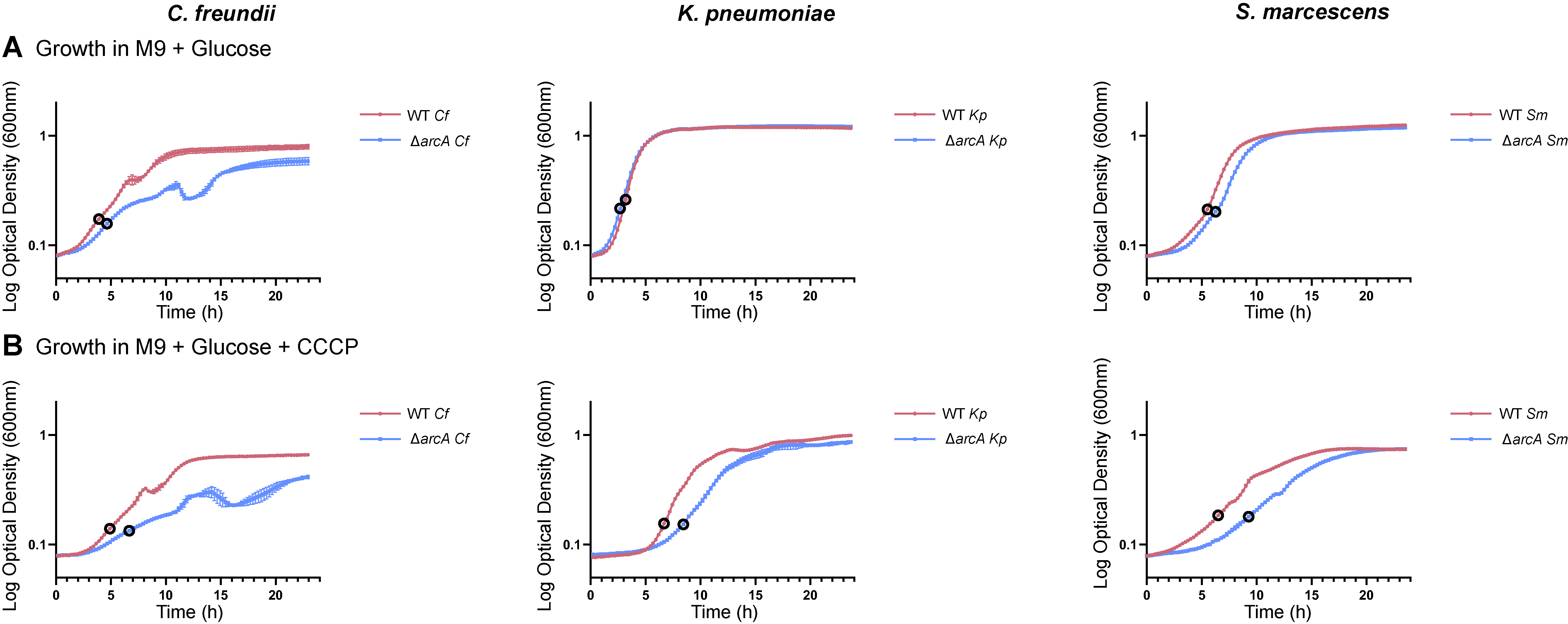

Supplement: Figure S5 — Sampling points for targeted metabolomics. [file mbio.01448-23-s0006.tif]

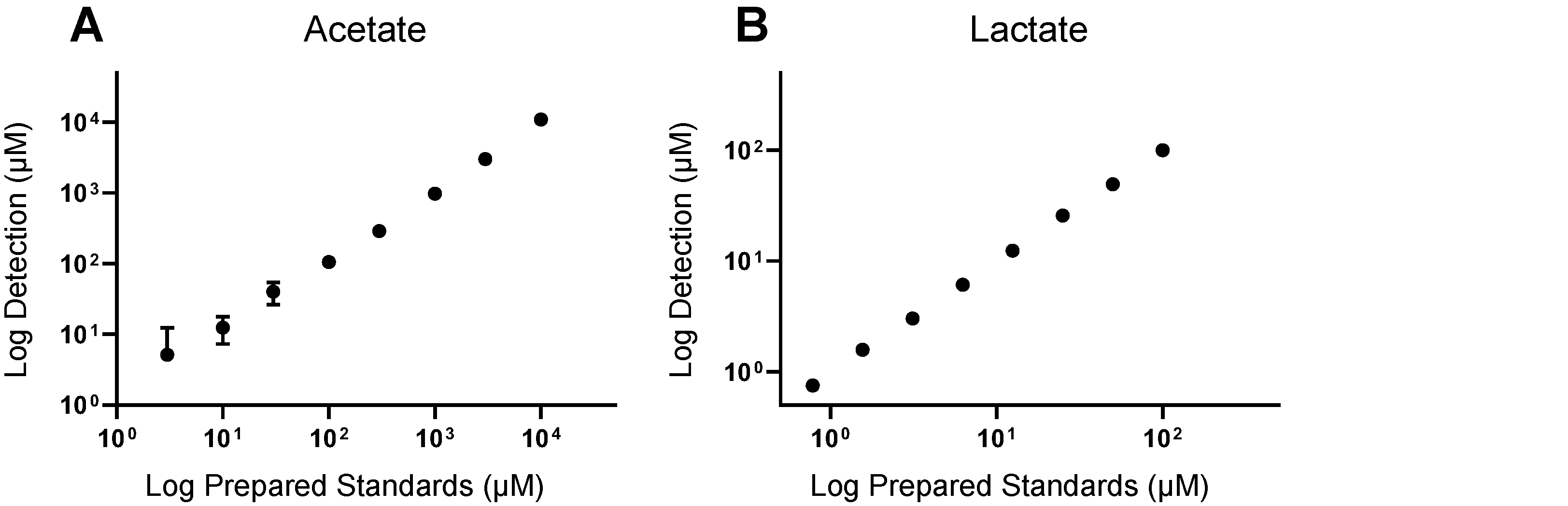

Supplement: Figure S6 — Acetate and lactate standards for LC-MS. [file mbio.01448-23-s0007.tif]
