# Supplementary material for: Conserved metabolic regulator ArcA responds to oxygen availability, iron limitation, and cell envelope perturbations during bacteremia
Source: mBio. 2023 Sep 8;14(5):e01448-23. doi: 10.1128/mbio.01448-23 (PMC10653796; doi:10.1128/mbio.01448-23)
Supplement: Supplemental Text — Supplemental Materials and Methods and figure legends. [file mbio.01448-23-s0008.docx]

**SUPPLEMENTAL MATERIALS and METHODS**

**Genetic Reversion and Complementation**

The *C. freundii* and *S. marcescens* Δ*arcA*::*npII* alleles were reverted to wild-type via recombineering to confirm that phenotypes observed in the *arcA::npt* mutant constructs are due to loss of *arcA*. Primers were designed to amplify the portion of the *arcA* gene replaced by the antibiotic resistance cassette in the Δ*arcA*::*npII* mutants with the same homologous ends as the inserts from the first round of Lambda red recombineering. *C. freundii* and *S. marcescens* *arcA* mutant strains were transformed with the resultant *arcA*-containing PCR products. Recovery of cells was performed in LB without selection at 30°C. Transformants were passaged overnight in LB at 30°C serially for two (*S. marcescens*) or three (*C. freundii*) days to enrich for revertants since *arcA* mutants were observed to grow more slowly relative to wild-type cells. Cultures were plated each day on LB without selection, and colonies were screened based on reversion to the wild-type colony size as *arcA* mutants exhibit a small colony phenotype. Reversion was confirmed by observing loss of kanamycin resistance and Sanger sequencing of PCR products amplified from the *arcA* locus.

The *K. pneumoniae* Δ*arcA*::*npII* mutant was complemented *in trans* with pBBR1MCS-5. Primers ANB21F and ANB21R and were used to amplify the *arcA* ORF and 539 base pairs upstream of the start of the gene with Easy A polymerase (Agilent). The PCR product and pBBR1MCS-5 parent plasmid were separately digested with SacI and XbaI. Ligation of the two digested fragments was achieved with T4 DNA ligase (NEB) followed by electroporation into *E. coli* TOP10 (Thermo Fischer). Plasmid construction was confirmed by Sanger sequencing. KPPR1 *ΔarcA::npII* was transformed with complementation plasmid by electroporation the complementation or empty vector control plasmids were maintained in the presence of gentamicin (10 µg/ml).

**Gene expression**

1.0mL of treated and untreated culture were added directly to 2mL of RNA protect solution (Qiagen), and RNA was extracted with the RNeasy Mini Kit (Qiagen) following manufacturer’s instructions. RNA samples were treated with RQ1 DNase (Promega) and repurified with the RNeasy Mini Kit (Qiagen). cDNA was generated with iScript cDNA Synthesis Kit (Bio-Rad) and diluted 1:10 with water. In untreated conditions, gene expression was compared to the wild-type strain following log transformation, and significance was determined via a one-sample t-test with a null hypothetical value of zero. Expression of each gene was compared between untreated and polymyxin B conditions, and significance was determined by comparing the wild-type strain with the mutant and complemented strains with Dunnett's multiple comparisons test.

**Metabolite quantification**

Aerobic and anaerobic overnight cultures were normalized by OD600, washed and resuspended in PBS, and subcultured into M9 media with carbonylcyanide-*m*-chlorophenylhydrazone (CCCP) at 15µM (*C. freundii*), 20µM (*K. pneumoniae*) and 25µM (*S. marcescens*). 300µL from each culture was added to five wells in a honeycomb plate. Plates were incubated on a Bioscreen-C plate reader with the following settings: 37°C, intermediate continuous shaking, OD600 measurement every 15 minutes. OD_600_ readings were monitored in real time via the Bioscreen-C plate reader. Upon reaching early exponential phase, 300uL was removed from two wells for each strain and condition and immediately transferred to ice. An aliquot of each sample was removed for CFU enumeration before cells were pelleted in a 4°C microcentrifuge. The supernatant was transferred to a new tube and immediately stored at -80°C. Supernatant samples were processed by the University of Michigan Metabolomics Core to quantify acetate and lactate. Growth is reported as the average of the unsampled wells for each genotype and condition.

Short chain fatty acids (SCFAs), including acetate, were measured using a modified version of a previously described protocol. SCFAs in the sample supernatant were derivatized using 3-nitrophenylhydrazine and an EDAC-6% pyridine solution. Samples were analyzed via LC-MS alongside acetate controls ranging from 3µ to 3000µM using an Agilent (Santa Clara, CA) 1290 LC coupled to an Agilent 6490 triple quadrupole MS. The chromatographic column was a Waters (Milford, MA) HSS T3, 2.1 mm x 100 mm, 1.7 µm particle size. Quantitation was performed using Agilent MassHunter Quantitative Analysis software version 8.0 by measuring the ratio of peak area of the 3-NPH derivatized SCFA species to its closest internal standard.

Lactate quantification was performed starting with the addition of an extraction solvent containing ^13^C Lactate to each supernatant sample. Following a series of mixing and centrifugation, supernatant was collected and dried using a nitrogen blower. Samples were reconstituted alongside a series of calibration standards. Ion pairing reverse phase LC-MS analysis was then performed using an Infinity Lab II UPLC coupled with a 6545 QTof mass spectrometer (Agilent Technologies, Santa Clara, CA) and a JetStream ESI source in negative mode. Chromatographic separation was performed on an Agilent ZORBAX RRHD Extend 80Å C18, 2.1 × 150 mm, 1.8 μm column with an Agilent ZORBAX SB-C8, 2.1 mm × 30 mm, 3.5 μm guard column. Data were processed using MassHunter Quantitative analysis version B.07.00.

**Lactate dehydrogenase measurement**

Bacteria were cultured as described for metabolite quantification and early exponential phase cells were collected via centrifugation at 4°C. The supernatant was removed, and cells were washed once then resuspended in PBS at 4°C prior to sonication. Cells were lysed by sonication with a taper microtip Z192740-1EA (Sigma-Aldrich) with the following protocol: 1 min. 40s. sonication at 40% amplitude with 4 s. bursts divided by 10 s. pauses to avoid overheating. Lactate dehydrogenase was measured in triplicate from cleared lysates with the Amplite® Fluorimetric D-Lactate Dehydrogenase (LDH) Assay Kit from AAT Bioquest per the manufacturer’s instructions. Fluorescence (excitation: 540nm; emission: 590nM) was measured after one hour incubation at room temperature protected from light with a Synergy H1 plate reader. LDH was quantified in samples based on standards ranging from 1µM/mL LDH to 200µM/mL. LDH concentration per 1 x 10^9^ cells was subsequently normalized based on CFU enumeration and compared for each strain between untreated and CCCP-treated conditions. Significance was determined by comparing LDH levels for strains in untreated and treated conditions using Šídák's multiple comparisons test.

***In silico* analyses**

ArcA amino acids sequences (n=419) from 418 *Enterobacterales* species were collated from BV-BRC (**File S1**). A multi-sequence alignment was generated with MUSCLE via EMBL-EBI. An ArcA predicted structure AF-P0A9Q1-F1 from Alpha Fold in agreement with a previously partially solved structure was retrieved via UniProt to serve as a template for conservation mapping. Consurf calculated conservation scores from the multiple sequence alignment based on the sequence extracted from the predicted structure via a JTT evolutionary model (**File S2**). A phylogenetic tree was constructed from the provided alignment via Neighbor Joining with ML distance. Rate4site then calculated residue specific conservation scores with Bayesian method providing confidence intervals, which were mapped onto the predicted ArcA structure with visualization of this projection provided by PyMOL. The ArcA amino acid sequences of *C. freundii* UMH14, *E.* coli CFT073, K*. pneumoniae* KPPR1, and *S. marcescens* UMH9 were aligned with Clustal Omega. A percent identity matrix was generated to calculate pairwise percent identities for all possible combinations of the four species. The output of amino acid alignment between all four species was then examined to assess conservation. Similarity of non-conserved residues was defined according to set parameters with a Gonnet PAM 250 matrix score of >0.5 signifying “strongly similar” and a score <0.5 and >0 for “weakly similar” residues.

ArcA binding sequences in the promoters of *acs*, *astC*, *fadE*, *feoB*, *lldP*, *putP*, and *ugpB* from *E. coli* K-12 MG1655 were used as the input motif with which to scan the promoters of the same genes in *C. freundii*, *K. pneumoniae*, and *S. marcescens*. Sequences identified by FIMO Version 5.5.1 from MEME Suite were reported as potential ArcA binding sequences when *p-*values and *q-*values (false discovery rate) were both ≤0.05. Nucleotides of the *E. coli* sequence and the sequences of the other species were compared to assess homology. Putative direct repeats were mapped onto the proposed ArcA sequences based on the coordinates reported in the *E. coli* sequences.

**SUPPLEMENTAL FIGURES and TABLES**

**Fig. S1: A majority of ArcA residues are evolutionarily conserved across Order *Enterobacterales*.** Consurf was utilized to map an alignment of 419 ArcA amino acid sequences from 418 species in Order *Enterobacterales* onto a predicted structure of ArcA from AlphaFold. Evolutionary rates were calculated via the JTT model of substitution based on an alignment from MUSCLE. A phylogenetic tree was constructed from the alignment using Neighbor Joining with ML distance. Rate4site then calculated residue specific conservation scores with Bayesian method providing confidence intervals. Residue conservation scores were visualized on a structure of ArcA with pyMOL (**Fig. 1B**).

**Fig. S2: ArcA is highly conserved at the amino acid level.** The amino acid sequences of ArcA from *C. freundii*, *E. coli, K. pneumoniae*, and *S. marcescens* were aligned with Clustal Omega (21988835). Symbols beneath the alignment are representative of the following: asterisk (*) – amino acid conserved across all species; colon (:) – strongly similar – Gonnet PAM 250 matrix score >0.5; period (.) – weakly similar – Gonnet PAM 250 matrix score <0.5 and >0; space ( ) – residues not conserved based on amino acid properties. The red arrow at position 54 identifies the conservation of an aspartate residue in all four species which is known participate in the canonical ArcB-ArcA phosphorelay.

**Fig. S3: Growth of bacterial strains in M9 + glucose + casamino acids in anaerobic conditions.** Strains were cultured overnight in LB under anaerobic or aerobic conditions and then normalized based on OD­_600_ in PBS_­­­_. M9 minimal medium with 0.4% glucose and 0.1% casamino acids was inoculated with normalized overnight cultures and incubated in an anaerobic chamber. OD_600­_ was measured with a plate reader every 10 minutes. Results are representative of three independent experiments. Each strain was grown in triplicate, and the average with standard deviation was plotted above.

**Fig. S4: Putative ArcA binding sequences of *C. freundii* and *S. marcescens*.** FIMO was utilized to search for ArcA binding boxes from *E. coli* MG1655 in the promoter regions of the seven genes evaluated in the *K. pneumoniae* expression studies. Sequences having a *p-*value and *q­-*value at or below 0.05 were considered significant and analyzed more closely. **(A)** In the promoters of 7/7 *C. freundii* genes, a putative ArcA binding sequence was identified. **(B)** In the promoters of 4/7 *S. marcescens* genes, a putative ArcA binding sequence was identified. Red nucleotides were loci not conserved between the *E. coli* and the corresponding species’ nucleotide sequences. Direct repeats within sequences were labeled based on coordinates of direct repeats within corresponding promoters of *E. coli* genes and are denoted by blue boxes.

**Figure S5: Sampling points for targeted metabolomics**. Overnight cultures incubated aerobically in LB were inoculated into M9 minimal medium with 0.4% glucose without (**A**) or with (**B**) CCCP (*C. freundii* - 15µM CCCP, *K. pneumoniae* - 20µM CCCP, *S. marcescens* - 25µM CCCP). Wild-type and Δ*arcA* cultures were incubated at 37°C in aerobic conditions and growth was tracked via OD_600_ by a plate reader every 15 minutes in quintuplet replicates. Upon reaching early exponential phase, cultures from two wells for each strain and each strain were removed for processing. The remaining three wells with culture were allowed to continue to grow. Sampling points are representative by open, black circles. Supernatants from the removed cultures were used in targeted metabolomics to quantitate acetate and lactate as described in the methodology section (**Fig. 7**).

**Figure S6:** **Acetate and lactate standards for LC-MS**. Acetate and lactate were measured via separate LC-MS protocols as described in methodology. Standards of acetate and lactate were included alongside experimental samples to allow for metabolite quantification. **(A)** Prepared acetate controls ranging from 3µM to 3000µM were quantified in duplicate using an Agilent 1290 LC coupled to an Agilent 6490 triple quadrupole MS. **(B)** Prepared lactate controls ranging from 0.7813µM to 100µM were quantified in duplicate on an Infinity Lab II UPLC coupled with a 6545 QTof mass spectrometer using a JetStream ESI source in negative mode. The averages of each control are plotted with standard deviation.
