# Supplementary material for: Conserved metabolic regulator ArcA responds to oxygen availability, iron limitation, and cell envelope perturbations during bacteremia
Source: mBio. 2023 Sep 8;14(5):e01448-23. doi: 10.1128/mbio.01448-23 (PMC10653796; doi:10.1128/mbio.01448-23)
Supplement: Table S1 — Primers. [file mbio.01448-23-s0009.docx]

**Table S1: Oligonucleotide primers utilized in this study**

| **Primer Set** | **Forward Primer (5’-3’)** | **Reverse Primer (5’-3’)** | **Description** |
| --- | --- | --- | --- |
| ***C. freundii* UMH14** | | | |
| ANB0009 | ATGCAGACCCCGCACATTCTTATCGTTGAAGACGAGTTAGTAACACGCAATGTAGGCTGGAGCTGCTTCG | AATCCTGCAGGTCGCCGCAGAAACGGTAGCCTTCACCGTGAATGGTGGCGCATATGAATATCCTCCTTAGT | λ-red kanamycin cassette insert for mutagenesis |
| ANB0012 | CTACCCACGACCAAGCTAATG | CAGAGTGACGAGTTTCGCTTAT | Confirmation of Δ*arcA* mutation |
| ANB0054 | ATGCAGACCCCGCACATTCTTATCGTTGAAGACGAGTTAGTAACACGCAACACGTTGAAAAGCATTTTCG | AATCCTGCAGGTCGCCGCAGAAACGGTAGCCTTCACCGTGAATGGTGGCGATAATTTCTGGCGTATCCGG | *λ*-red insert for Δ*arcA*::*nptII* reversion |
| ANB0055 | CGGCATGTCTTAGCCTGTTAT | TCCCAGCCATTGAACTTGTAG | Screening for Δ*arcA*::*arcA* revertant |
| ***E. coli* CFT073** | | | |
| ANB0017 | TCAGGCAGGTCAGGGACTTTTGTACTTCCTGTTTCGATTTAGTTGGCAATTTGTAGGCTGGAGCTGCTTCG | CGATGAATTACGTATCTGGAAATAAGATAGAAAAATAAAAACGGCGCTAAACATATGAATATCCTCCTTAGT | λ-red kanamycin cassette insert for mutagenesis |
| ANB0018 | TGTTGTTGACGTTGATGGAAAG | GACCCGTAATATCGACTGGTATG | Confirmation of Δ*arcA* mutation |
| ***K. pneumoniae* KPPR1** | | | |
| ANB0010 | ATGCAGACCCCGCACATTCTTATCGTTGAAGACGAGTTGGTAACACGCAATGTAGGCTGGAGCTGCTTCG | TTATTCCTGCAGGTCGCCACAGAAACGGTAGCCTTCACCGTGAATGGTAGCATATGAATATCCTCCTTAGT | λ-red kanamycin cassette insert for mutagenesis |
| ANB0013 | GGGACTTTGGTACTTCCTGTT | CGTGGACTGGTATGCGTTAT | Confirmation of Δ*arcA* mutation |
| ANB0021 | CCCCTCTAGAAATTGCGTTTTCTTACCC | CCCCGAGCTCGCTGAGCGTCCTGATGTTTTT | For cloning *arcA* into pBBR1MCS-5 |
| M13 | CGCCAGGGTTTTCCCAGTCACGAC | CAGGAAACAGCTATGAC | Confirmation of pBBR1MCS-5 + *arcA* construct |
| ANB0040 | CGCAGAGTACATGGCTTACA | TTACCGTTAACGACCAGATGAC | For qPCR of *gapA* |
| ANB0029 | CAATCCATAACCGACCCTGATAC | TGCGAAGGAGGTGTTCTTTAC | For qPCR of *acs* |
| ANB0026 | CTATGGCCTGTTCAAGGAGAT | GCCAGGTTACTGAGGGTTT | For qPCR of *astC* |
| ANB0022 | GCTAACGACTTCGCCATCA | TCAAGTCCGCCATACTCTTTC | For qPCR of *fadE* |
| ANB0030 | GTGACCGTTGAGCGTAAAGA | AGATGGTGGTCAGGGAGTAG | For qPCR of *feoB* |
| ANB0028 | TCGGATCTGTTCCGCTTTAC | CACGGGATCATCCAGGTTAAA | For qPCR of *lldP* |
| ANB0023 | GTGGTCCTGTTCTCGGTAATG | AACTGCTTCCAGACGATCAC | For qPCR of *putP* |
| ANB0027 | TGCTCGAAGAGATGAACAAGAA | GGACGAGGCAGTGGTAATG | For qPCR of *ugpB* |
| ***S. marcescens* UMH9** | | | |
| ANB0008 | TATGCAGACCCCGCACATTCTGATTGTCGAAGACGAGTTAGTCACTCGTATGTAGGCTGGAGCTGCTTCG | GCCGTGAATGGTGGCGATGATTTCCGGCGTGTCCGGCGTCGATTCGAAGTCATATGAATATCCTCCTTAGT | *λ*-red kanamycin cassette insert for mutagenesis |
| ANB0011 | TCTCTGCCCTCCTCTAAGAAA | CGTGGGCTGAGACGAAATAA | Confirmation of Δ*arcA* mutation |
| ANB0044 | TATGCAGACCCCGCACATTCTGATTGTCGAAGACGAGTTAGTCACTCGTAACACCCTGAAGAGCATTTTC | GCCGTGAATGGTGGCGATGATTTCCGGCGTGTCCGGCGTCGATTCGAAGTGTTTGCGAATGCGACGGATGG | *λ*-red insert for Δ*arcA*::*nptII* reversion |
| ANB0045 | TCGGCACACGCTGTTATATT | ACCGTTGAACTTGTAGCTCTC | Screening for Δ*arcA*::*arcA* revertant |
